# Current, emerging, and potential therapies for non-alcoholic steatohepatitis

**DOI:** 10.3389/fphar.2023.1152042

**Published:** 2023-03-30

**Authors:** Zhen Yang, Lin Wang

**Affiliations:** Department of Hepatobiliary Surgery, Xi-Jing Hospital, The Fourth Military Medical University, Xi’an, China

**Keywords:** non-alcoholic fatty liver disease, lipid peroxidation, non-alcoholic steatohepatitis, metabolic homeostasis, ferroptosis, targeted therapeutics, the gut microbiome

## Abstract

Non-alcoholic fatty liver disease (NAFLD) has been identified as the most common chronic liver disease worldwide, with a growing incidence. NAFLD is considered the hepatic manifestation of a metabolic syndrome that emerges from multiple factors (e.g., oxidative stress, metabolic disorders, endoplasmic reticulum stress, cell death, and inflammation). Non-alcoholic steatohepatitis (NASH), an advanced form of NAFLD, has been reported to be a leading cause of cirrhosis and hepatic carcinoma, and it is progressing rapidly. Since there is no approved pharmacotherapy for NASH, a considerable number of therapeutic targets have emerged with the deepening of the research on NASH pathogenesis. In this study, the therapeutic potential and properties of regulating metabolism, the gut microbiome, antioxidant, microRNA, inhibiting apoptosis, targeting ferroptosis, and stem cell-based therapy in NASH are reviewed and evaluated. Since the single-drug treatment of NASH is affected by individual heterogeneous responses and side effects, it is imperative to precisely carry out targeted therapy with low toxicity. Lastly, targeted therapeutic agent delivery based on exosomes is proposed in this study, such that drugs with different mechanisms can be incorporated to generate high-efficiency and low-toxicity individualized medicine.

## 1 Introduction

In general, chronic liver disease (CLD) comprises drug-induced liver disease, alcoholic liver disease, non-alcoholic fatty liver disease (NAFLD), viral hepatitis, liver cirrhosis, and liver cancer (Tomedi et al., 2018). NAFLD is the most common chronic liver disease, encompassing a spectrum of liver injuries ranging from fatty liver disease to liver cirrhosis (Setiawan et al., 2016; Younossi et al., 2018). The prevalence of NAFLD has been increasing (25.24% in 2015 to 29.38% in 2021) (Younossi et al., 2016; Liu et al., 2022), accounting for 45.8% of the total causes of death from chronic liver disease (Golabi et al., 2022). Non-alcoholic steatohepatitis (NASH) refers to an advanced form of NAFLD, and it is characterized by the presence of hepatic steatosis with hepatocellular injury (hepatocyte ballooning) and inflammation, which is one of the main causes of cirrhosis and hepatocellular carcinoma (Goldberg et al., 2017).

Overnutrition is the initiating event of NAFLD, resulting in the expansion of fat depots and accumulation of ectopic fat. The imbalance of lipid metabolism leads to the production of toxic metabolites in the setting of insulin resistance, thus resulting in oxidative stress and endoplasmic reticulum (ER) stress, lipid peroxidation and inflammasome activation, apoptotic cell death, and tissue fibrosis and regeneration (Sanyal, 2019). NAFLD is affected by a multitude of pathogenic pathways (e.g., metabolic disorders, hormones secreted from the adipose tissue, overnutrition, oxidative stress, and genetic and epigenetic factors) (Buzzetti et al., 2016). The pathogenesis and clinical manifestations of NAFLD are highly heterogeneous, which may be correlated with different pathogenic factors (Alonso et al., 2017). Furthermore, disease progression and response to treatment are not universally identical. Nutrition and energy balance takes on a critical significance in the development of NAFLD and NASH. Diet quality is highly important in the management and prevention of NAFLD (Ma et al., 2018). Adherence to a Mediterranean dietary pattern and energy restriction exhibit a favorable effect on the reduction of liver fat accumulation and steatohepatitis (Trovato et al., 2015; European Association for the Study of the Liver EASL et al., 2016). Physical activity is conducive to the prevention of NAFLD and ameliorates fatty liver (Sung et al., 2016; Kwak et al., 2017). Weight loss is capable of significantly ameliorating hepatic steatosis and even fibrosis (Vilar-Gomez et al., 2015). Lifestyle intervention contributes to the prevention of the progression of NAFLD to NASH, and it is fundamental to other therapies. Metformin refers to an antihyperglycemic agent with lipid-lowering effects. However, there is insufficient evidence to support metformin’s therapeutic effect on NAFLD (Iogna Prat and Tsochatzis, 2018). Even though no convincing evidence implicates that improvement in hepatic histology emerges under statin therapy, patients with NASH have a marked reduction in the risk of major cardiovascular events (Dongiovanni et al., 2015; Tziomalos et al., 2015). Bile acid metabolism is closely correlated with NASH; perhaps bile salt-based therapies have a significant treatment landscape (Donkers et al., 2019; Okushin et al., 2020).

There has not been any approved treatment for NASH. An increasing number of novel targets and strategies have emerged with the development of the pathogenesis of NASH. Notably, NASH has a rapid progression, and there is little evidence that NASH is always secondary to NAFLD, such that existing drug research and development is focused on NASH. In this review, the focus is placed on the summary of therapeutic potential and utility of metabolic regulation, the gut microbiome, antioxidant, microRNA, inhibiting apoptosis, targeting ferroptosis, and stem cell-based therapy in the progression of NASH. Furthermore, the potential and value of exosome-mediated targeted therapeutic agent delivery in the treatment of NASH are evaluated.

## 2 Therapy with regulating metabolism in NASH

**FIGURE 1:**
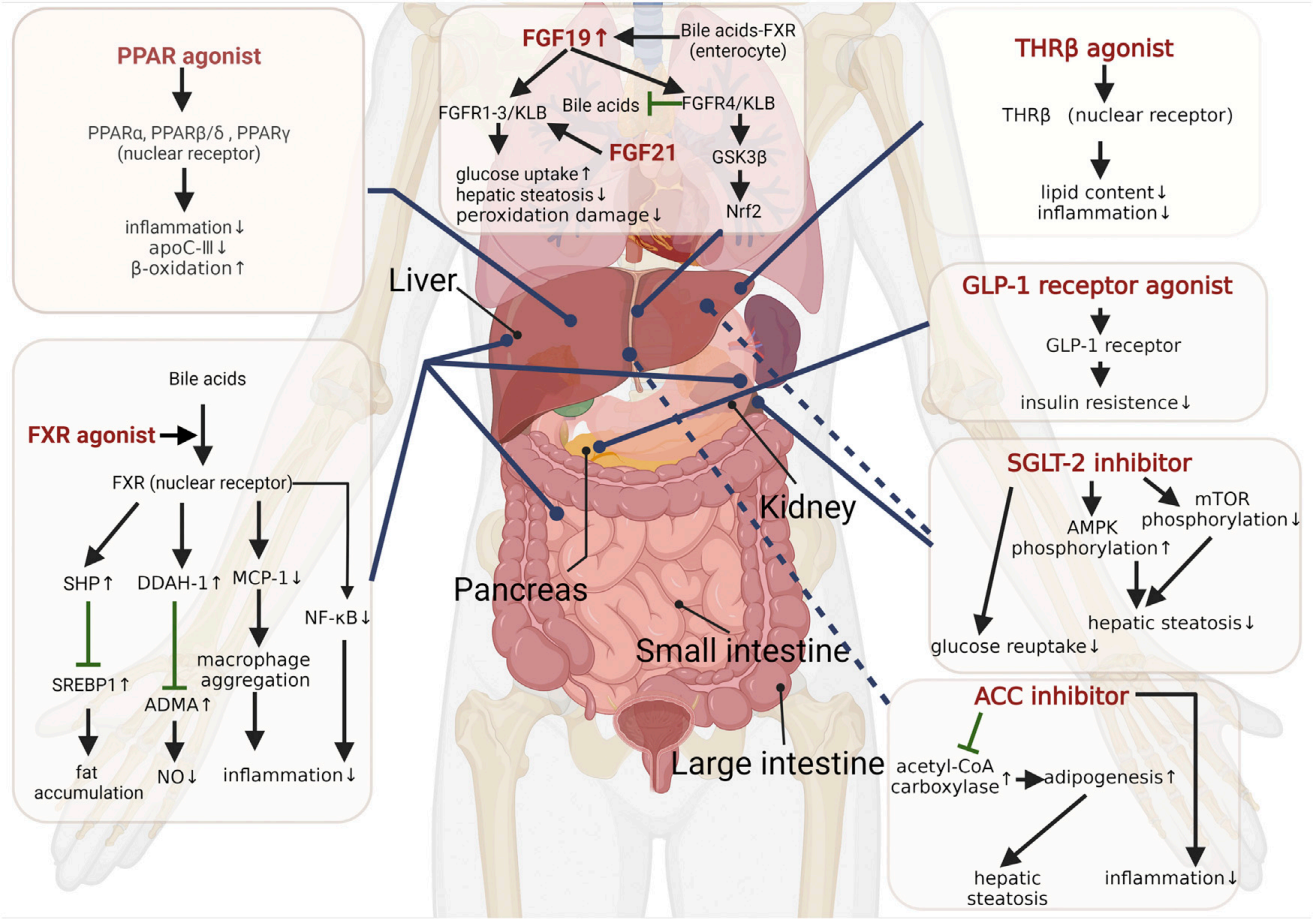
Targets for correcting metabolic disorders in NASH.

Generally, NASH is considered a hepatic manifestation of the metabolic syndrome, and metabolic disorders are involved throughout the course of NASH (Neuschwander-Tetri and Caldwell, 2003; Yki-Järvinen, 2014; Masoodi et al., 2021; Tilg et al., 2021). Maintaining and improving metabolic homeostasis has been proven as a therapeutic strategy for NASH. Several therapies for improving metabolism are shown in the following (Figure 1).

### 2.1 Peroxisome proliferator-activated receptor agonists

Peroxisome proliferator-activated receptors (PPARs), a group of nuclear receptors and heterodimers with retinoid X receptors, are capable of regulating multiple metabolic processes through transcription. PPARs comprise three subtypes (i.e., PPARα, PPARβ/δ, and PPARγ), with different targeting ligands, and they are present in several tissues at different levels. PPARs exhibit vital regulatory effects as lipid sensors in response to fatty acid transport, gluconeogenesis, and β-oxidation (Lalloyer and Staels, 2010; Fougerat et al., 2020).

PPARα is mostly expressed in the liver and brown adipose tissue, which improves lipid metabolism by regulating fatty acid transport and β-oxidation. The activation of PPARα can increase NASH-related intestinal permeability and improve liver inflammatory injury (Velkov, 2013; Régnier et al., 2020). Moreover, the activation of PPARα attenuates plasma levels of proinflammatory cytokines, such as IL-1, IL-6, and TNF, and improves triglyceride (TG) metabolism *via* upregulating the expression of lipoprotein lipase (Mansouri et al., 2008) and suppressing hepatic apolipoprotein C-Ⅲ (apoC-Ⅲ) secretion (Qu et al., 2007). The histological improvement of NASH is correlated with an increased expression of PPARα and its target gene, but not PPARβ/δ and PPARγ (Francque et al., 2015). PPARγ is mainly distributed in the immune system and adipose tissue, where its functions are immunomodulation and ameliorating insulin sensitivity (Croasdell et al., 2015). The peroxisome proliferator-activated receptor-*γ* coactivators (PGC1s) have the potential for treating NAFLD as regulators of mitochondrial metabolism and immune responses (Piccinin et al., 2019). Binding of β-arrestin-1 to PPARγ suppresses adipogenesis and inflammation, and ameliorates systemic insulin sensitivity (Zhuang et al., 2011). PPARβ/δ also has the function of regulating glucose and lipid metabolisms. PPAR agonists that have made progress in clinical studies are summarized and discussed as follows.

Pemafibrate (K-877) is a novel selective PPARα modulator that improves dyslipidemia, plasma transaminase level, and the pathological condition of NASH in animal trials (Honda et al., 2017). Pemafibrate regulates glucose oxidation and facilitates fatty acid oxidation by inducing the expression of key target genes, including 3-hydroxymethylglutaryl-CoA synthase 2 (HMGCS2) and pyruvate dehydrogenase kinase 4 (Sasaki et al., 2019). Clinical research studies show that pemafibrate can effectively and safely decrease plasma TG, VLDL cholesterol, remnant cholesterol, and apoC-III levels (Das Pradhan et al., 2022; Ginsberg et al., 2022). A previous study indicated that pemafibrate significantly decreased magnetic resonance elastography-based liver stiffness without reducing liver fat content (Nakajima et al., 2021). The adverse effects of pemafibrate include adverse renal events and venous thromboembolism (Das Pradhan et al., 2022). Further studies are required to determine whether pemafibrate can alleviate NASH progression and repair histological damage.

Pioglitazone (a synthetic ligand of PPARγ) which is used clinically for the treatment of type 2 diabetes mellitus (T2DM) mitigates steatosis, inflammation, and fibrosis in subjects with NASH (Belfort et al., 2006; Aithal et al., 2008); however, it increases the patient’s weight and risk of bladder cancer (Sanyal et al., 2010; Tang et al., 2018). Rosiglitazone, a selective PPARγ activator, did not ameliorate NASH, but inhibited lipogenesis in the rosiglitazone therapy (FLIRT 2) extension trial (Ratziu et al., 2010).

Elafibranor (GFT505) is a PPARα/δ dual agonist that avoids problems, such as weight gain, edema, and induction of heart failure, associated with the activation of PPARγ. A phase Ⅱ trial showed that elafibranor improved NASH without deteriorating hepatic fibrosis (Ratziu et al., 2016). Nevertheless, another phase Ⅲ trial reported that elafibranor failed to reach the predefined histological endpoint since there was no significant difference in the rate of histological resolution (Ratziu et al., 2016).

Saroglitazar (a PPARα/γ dual agonist) is an anti-metabolic syndrome drug developed locally in India by the company named Zydus Cadila in 2001, which can improve insulin sensitivity, lipid, and glycemic parameters (Gowda et al., 2022). Saroglitazar ameliorates steatohepatitis and lowers lipid levels in diet-induced mice (Kumar et al., 2020). It improves histological features and reduces the liver fat content in NASH models (Jain et al., 2018). A phase Ⅱ trial demonstrated that saroglitazar significantly reduced serum ALT levels, insulin resistance, dyslipidemia, liver fat content, markers of hepatocyte injury, and fibrosis in NASH patients (Gawrieh et al., 2021). Saroglitazar administered to NASH patients resulted in improvement in histological changes (Siddiqui et al., 2021). Further investigation is required to determine whether it can reach the intended histological endpoint of NASH resolution.

The activation of individual receptors of PPARs has a limited effect, and the widespread activation of PPARs may have a more effective and radical therapeutic potential for NASH through multiple pathological mechanisms. Lanifibranor (IVA337) is a pan-PPAR agonist with moderate and balanced effects on PPARα (on hepatocytes), PPARδ (on macrophages), and PPARγ (on stellate cells) that can ameliorate all aspects of NAFLD (Lefere et al., 2020). A phase Ⅱb trial involving patients with active NASH showed that lanifibranor remarkably improved NASH without worsening fibrosis (Francque et al., 2021). The adverse effects of lanifibranor include diarrhea, nausea, anemia, and weight gain (Francque et al., 2021). The aforementioned findings provided support for the phase Ⅲ clinical trial of lanifibranor.

### 2.2 Farnesoid X receptor agonists

The farnesoid X receptor (FXR) refers to a nuclear receptor stimulated by bile acids (BAs) distributed in the liver, kidneys, intestines, and adrenal glands. It has the capability of maintaining metabolic homeostasis by decreasing liver fat accumulation through the FXR-SREBP1 signaling pathway (Schmitt et al., 2015; Liu et al., 2020) and regulating glucose metabolism (Ma et al., 2006). The FXR is also expressed in liver resident macrophages, Kupffer cells, natural killer cells, and dendritic cells and plays an immunomodulatory role (Fiorucci et al., 2022). The latest study found that FXR inhibition can prevent SARS-CoV-2 infection by reducing angiotensin-converting enzyme 2 (ACE2) (Brevini et al., 2022). Nevertheless, activating the FXR selectively inhibits a hepatic inflammatory reaction mediated by NF-κB and impairs the aggregation of macrophages into body tissues by downregulating monocyte chemoattractant protein-1 (MCP-1) levels (Wang et al., 2008; Li et al., 2015). An FXR agonist is capable of inhibiting asymmetric dimethylarginine (ADMA) accumulation through activating dimethylarginine dimethylaminohydrolase-1 (DDAH-1) expression, thereby increasing the levels of endogenous nitric oxide that regulates NASH progression (Hu et al., 2006; Cunningham et al., 2021). As revealed by a recent study, withaferin A as a dual liver X receptor-α (LXR-α) and FXR agonist ameliorated NAFLD-related hepatitis and liver fibrosis through multiple pathways, including inhibiting NF-κB and TGF-β signaling (Shiragannavar et al., 2023). Accordingly, FXR agonists have become a focus of research for NASH treatment.

Obeticholic acid (OCA), a derivative of chenodeoxycholic acid (the major component of human bile acid), serves as a potent agonist of the FXR with minimal activity to a G-protein-coupled bile acid receptor (Pellicciari et al., 2004). OCA suppresses the progression of NASH by protecting against disruption of the intestinal epithelial and gut vascular barriers (early events in the development of NASH) (Mouries et al., 2019). In clinical studies, OCA significantly improved histological features of NASH without worsening fibrosis in a higher proportion of patients than placebo (Neuschwander-Tetri et al., 2015; Younossi et al., 2019). However, OCA has not gained approval from the Food and Drug Administration (FDA) for treating NASH since its adverse effects outweigh its benefits (Mullard, 2020). The adverse effects comprise pruritus, elevated low-density lipoprotein cholesterol, and liver failure (Mullard, 2020). Tropifexor (LJN452) is a highly potent FXR agonist and induces a target gene following very low concentrations (Tully et al., 2017). Its clinical effect needs further investigation. There are several FXR agonists in the development of NASH treatment.

### 2.3 Thyroid hormone receptor β agonists

The thyroid hormone receptor (THR) is a member of the nuclear hormone receptor family that links and regulates transcription factors including two subtypes, THRα and THRβ. THRs are associated with multiple functions, such as development, differentiation, growth, and metabolism (Gionfra et al., 2019). THRβ is highly expressed in hepatocytes, and THRβ agonists decrease the levels of serum cholesterol, triglycerides, and intrahepatic lipid contents (Sinha et al., 2019). Interestingly, hepatic hypothyroidism occurs with the downregulated expression of deiodinase 1 and the upregulated expression of deiodinase 3 in patients with NAFLD (Bohinc et al., 2014). Resmetirom (MGL-3136), a liver-directed selective THRβ agonist, contributes to profound reduction in hepatic steatosis, plasma alanine aminotransferase activity, blood glucose, inflammatory, and fibrosis makers (Kannt et al., 2021). A phase Ⅱ clinical trial showed that resmetirom treatment distinctly reduced hepatic fat and improved hepatic inflammation in patients with NASH. There were no significant side effects among the groups, except for transient diarrhea and nausea (Harrison et al., 2019). VK2809 (MBD7811) undergoes the hepatic first pass effect to become VK2809A (a negatively charged THRβ agonist) and has high selectivity toward liver tissues (Erion et al., 2007). It has significant potential in the treatment of NASH. Based on the aforementioned findings, the THRβ agonist in patients with NASH and fibrosis is currently being investigated.

### 2.4 Glucagon-like peptide-1 receptor agonists

GLP-1 is an incretin hormone secreted by ileum cells after food intake. GLP-1 controls blood glucose by stimulating insulin secretion from islet *β* cells and inhibiting glucagon secretion from islet *α* cells. In addition to acting on the pancreas, existing research has suggested that GLP-1 improves peripheral insulin sensitivity, suppresses hepatic lipogenesis, delays gastric emptying, and reduces appetite (Knudsen and Lau, 2019). An increase in energy expenditure stimulated by GLP-1 receptor agonists through hypothalamic AMP-activated protein kinase (AMPK) pathways assists in weight reduction (Beiroa et al., 2014). Long-acting GLP-1 analogs have also been reported to alleviate hepatic steatosis (Armstrong et al., 2016). Tirzepatide is a novel dual GLP-1 and glucose-dependent insulinotropic peptide (GIP) receptor agonist, and its therapeutic efficacy in improving NASH has been reported in a clinical trial (Hartman et al., 2020). A previous study has confirmed that tirzepatide dose-dependently reduces NASH-related biomarkers and increases adiponectin (Hartman et al., 2020). The most common side effects of tirzepatide comprise emesis, nausea, decreased appetite, abdominal bloating, and diarrhea, which are considered mild to moderate in severity (Coskun et al., 2018). Semaglutide, another GLP-1 receptor agonist, exhibited powerful improvements against NASH without worsening fibrosis at a dose of 0.4 mg compared to placebo in a phase Ⅱ trial (Newsome et al., 2021). GLP-1 has served as a well-established target in diabetes treatment and is also a promising factor in treating NASH.

### 2.5 Fibroblast growth factor analogs

The family of fibroblast growth factor (FGF) peptides has 22 members and binds to five receptors (FGFR1–4 and FGFRL1). To be specific, FGF19 (a peptide directly targeting the liver) and FGF21 (a peptide mainly secreted by the liver) are promising targets for the treatment of NAFLD for their immunoregulatory effects, hepatoprotective properties, and regulatory capacity of metabolisms (Ocker, 2020). Alternatively, recent research has suggested that previously unappreciated FGF4 mitigates NAFLD and NASH through AMPK-caspase 6 signaling pathways (Song et al., 2022). The progress of FGF in NASH is described as follows.

FGF19 produced primarily in the ileum is significantly elevated after the meal *via* the BA-FXR pathway (Lundåsen et al., 2006; Somm and Jornayvaz, 2018). FGF19 is generally effective, requiring binding to FGFR1-4 and βKlotho (KLB) (Kurosu et al., 2007; Lin et al., 2007). It is capable of regulating hepatic protein synthesis and glucose metabolism mediated by the mitogen-activated protein kinase (MAPK) signaling pathway instead of the insulin-related pathway (Kir et al., 2011). FGF19 plays a cytoprotective role against ER stress by activating FGFR4-glycogen synthase kinase-3β (GSK3β)-nuclear factor E2-related factor 2 (Nrf2) signaling cascade (Teng et al., 2017). FGF19 and its analog modulate bile acid levels and ameliorate steatosis by inhibiting the hepatic expression of CYP7A1 and CYP27A1 which encodes the key enzymes for bile acid synthesis (Zhou et al., 2016). Aldafermin (NGM282) is a manufactured non-tumorigenic analog of FGF19 that binds to FGFR4/KLB. In a phase Ⅱ trial in NASH patients, aldafermin decreased liver fat content and the levels of serum markers, such as 7α-hydroxy-4-cholesten-3-one, alanine, and aspartate aminotransferases (Harrison et al., 2021a). More recently, aldafermin alleviated hepatic fibrosis without worsening NASH and had no apparent dose-dependent response (Harrison et al., 2022).

FGF21 activates multiple targets with binding FGFR1-3 and KLB (Kurosu et al., 2007), thereby initiating downstream signaling. FGF19 and FGF21 exhibit similar physiological effects. The downregulation of FGF21 is correlated with loss of hepatic fat following high-protein diet, and FGF21 may serve as the biomarker of metabolic improvement (Markova et al., 2017). FGF21 can improve PPARα-related lipid metabolism (Badman et al., 2007) while regulating adiponectin-mediated energy metabolism and insulin sensitivity in the liver (Lin et al., 2013). ER stress can upregulate FGF21 expression, such that ER stress can be ameliorated *via* the AMPK-sirtuin1 (SIRT1) pathway (Chau et al., 2010; Kim et al., 2015). FGF21 reduces hepatic steatosis and peroxidation damage in NASH by regulating the activation and oxidation of fatty acids in the liver (Fisher et al., 2014). Hence, FGF21 analog is expected to become a therapeutic agent for NASH. Pegbelfermin (BMS-986036), a PEGylated analog of FGF21, has been shown to safely reduce hepatic fat fraction in patients with NASH (Sanyal et al., 2019), as has efruxifermin (AKR001), another long-acting Fc-FGF21 fusion protein (Harrison et al., 2021b).

### 2.6 Sodium–glucose cotransporter-2 inhibitors

Sodium–glucose cotransporter-2 (SGLT-2) has been reported as a critical mediator of epithelial glucose transport in charge of the majority of glucose reuptake in the kidney (Rieg and Vallon, 2018). SGLT-2 inhibitors as new hypoglycemic agents improve blood glucose and lower weight by preventing SGLT-2 from renal reabsorption of sodium and glucose (Kaneto et al., 2016). An SGLT inhibitor can activate AMPK phosphorylation and inhibit the mammalian target of rapamycin (mTOR) phosphorylation, thereby improving hepatic steatosis (Luo et al., 2021a). The SGLT-2 inhibitor also exhibits cytoprotective properties, reduces hepatic lipid contents, and attenuates oxidative stress (Honda et al., 2016; Kabil and Mahmoud, 2018). Furthermore, the promotion of autophagy and reduction of ER stress and hepatocyte apoptosis are mediated by SGLT-2, alleviating the progression of NAFLD (Nasiri-Ansari et al., 2021). In a clinical study, empagliflozin (SGLT-2 inhibitor) significantly reduced hepatic fat content in patients with type 2 diabetes and NAFLD (Kuchay et al., 2018). Thus, SGLT-2 inhibitors have been proposed as treatment options for NASH.

### 2.7 Acetyl-CoA carboxylase inhibitors

Conversion of acetyl-CoA to malonyl-CoA mediated by acetyl-CoA carboxylase (ACC) is one of the vital steps of adipogenesis. The ACC inhibitor can improve fatty degeneration of the liver and insulin resistance, such that it is promising for the treatment of NASH (Harriman et al., 2016). PF-05221304, an ACC inhibitor that targets the liver, alleviates inflammation and fibrosis in NASH (Ross et al., 2020). However, PF-05221304 may elevate serum triglyceride levels by increasing SREBP1c expression and hepatic VLDL exportation (Kim et al., 2017). PF-05221304 coadministered with a diacylglycerol acyltransferase 2 (DGAT2) inhibitor is one pathway used to resolve this issue (Calle et al., 2021). DGAT2 is highly expressed in liver and adipose tissues, which catalyzes the terminal step of *de novo* lipid synthesis, particularly the esterification of fatty acids with diacylglycerol to form triglycerides. The combination of PF-05221304 and DGAT2 inhibitors dramatically lowers hepatic fat content with good safety and tolerability (Calle et al., 2021). Arachidonate 12-lipoxygenase (ALOX12) protects ACC1 from lysosomal degradation to promote the occurrence of NASH (Zhang et al., 2021b). IMA-1, identified as a small molecule, has been confirmed to be capable of interrupting the ALOX12–ACC1 axis *via* directly binding to a pocket in ALOX12, thereby preventing the progression of NASH with milder side effects (Zhang et al., 2021a). Additionally, ACC inhibitors can suppress the activation of HSCs to inhibit NASH-related fibrosis (Bates et al., 2020).

## 3 The gut microbiome as a therapeutic target for NASH

The mutualism between the gut microbiome and humans is a well-confirmed symbiosis, and the gut microbiome is beneficial to the health of the host (Sekirov et al., 2010; Lin and Zhang, 2017). There are different degrees of gut microflora dysbiosis in different stages of NAFLD. Some studies indicate that the overgrowth of *Bacteroides* and *Prevotella* genera may play a key role in the development of NASH (Mouzaki et al., 2013; Wong et al., 2013; Sobhonslidsuk et al., 2018). The gut microbiome affects liver disease and cancer by altering intestinal permeability and activating the innate immune system. The effect of the gut microbiome on host lipid metabolism is mediated by the metabolites generated from the gut microbiome (e.g., short-chain fatty acids, secondary bile acids, and trimethylamine), or by the components of bacteria themselves (e.g., lipopolysaccharides, peptidoglycans, and DNA), which interact with host hepatocytes through the portal vein (Ji et al., 2019). The disruption of gut–liver axis homeostasis leads to a variety of diseases, including NASH.

Probiotics are living microorganisms that provide health benefits to the host when consumed in sufficient amounts (Hill et al., 2014). Probiotics have the capability of increasing the diversity of intestinal flora and the relative abundance of beneficial bacteria, inhibiting lipid synthesis, reducing liver inflammation, and increasing antioxidant enzyme activity (Park et al., 2020; Luo et al., 2021b). Prebiotics are organic substances that selectively promote the metabolism and proliferation of beneficial bacteria without being digested and absorbed by the host, thereby improving the health of the host. Prebiotics possess high potential to ameliorate NASH, as do probiotics (Carpi et al., 2022). Fecal microbial transplantation (FMT) refers to the transplantation of functional microflora from the donor’s feces into the patient’s gut in an attempt to improve the original intestinal flora imbalance and rebuild the normal intestinal microecology, achieving the treatment of disease (Khoruts, 2018). Following FMT therapy, gut microbiota dysbiosis was corrected, the abundance of beneficial bacteria was increased, and steatohepatitis was attenuated in mice with high-fat diet (HFD) (Zhou et al., 2017). A clinical study has suggested that FMT improves intestinal permeability in patients with NAFLD, while there is no increase in insulin sensitivity or decrease in intrahepatic lipid content (Craven et al., 2020). The efficacy of FMT is closely related to the characteristics of the donors, especially the richness and diversity of fecal microbiota. Therefore, whether FMT can be used in the treatment of NASH still needs a large number of trials to provide evidence. Furthermore, several antibiotics are used to ameliorate gut microbiome dysbiosis associated with NASH. Rifaximin is a non-systemic antibiotic that is barely absorbed in the gastrointestinal tract and does not significantly affect the diversity of the gut microbiome (Kaji et al., 2017). However, the effect of rifaximin in the treatment of human NASH remains debatable (Cobbold et al., 2018; Jian et al., 2022). Triclosan and solithromycin are antibiotics that improve intestinal homeostasis and hepatic steatosis (Sumida and Yoneda, 2018; Sun et al., 2022). On one hand, antibiotics regulate the disorder of intestinal flora to achieve the purpose of treatment, and on the other hand, it leads to the occurrence of bacterial resistance and produce new flora disorders when used in the long-term. Antibiotics must be used with caution for treating NASH. In the following period, the gut microbiome will be a promising therapeutic target for NASH therapy.

## 4 Targeting oxidative stress in the treatment of NASH

Oxidative stress caused by ROS is an important contributing factor in the progression of NASH. As a powerful biological antioxidant, vitamin E has a protective effect on cell membranes and lipoproteins, preventing lipid peroxidation. Vitamin E reduces liver injury by ameliorating mitochondrial oxidative damage and inhibiting apoptosis (Soden et al., 2007). Studies have revealed that vitamin E reduced steatosis, lobular inflammation, and hepatocyte ballooning, but not fibrosis, in NASH (Sanyal et al., 2010). However, the safety of vitamin E has raised a concern as a result of multitudinous adverse effects, such as increasing all-cause mortality, incidence of prostate cancer, and risk of hemorrhagic stroke (Miller et al., 2005; Lippman et al., 2009; Schürks et al., 2010). Vitamin E as a hydrophobic compound is unable to translocate across the membrane into the cytoplasm and mitochondria, thereby limiting the antioxidant effect. Superoxide dismutase (SOD) plays an antioxidant role by catalyzing superoxide anions to hydrogen peroxide and molecular oxygen *in vivo*. rMnSOD is a recombinant humanized molecule that can cross the cell membrane to exhibit antioxidant effects (Borrelli et al., 2016). Meanwhile, rMnSOD has an excellent biodistribution in the liver and reduces portal pressure (Guillaume et al., 2013). It can safely and effectively halt the progression of NASH by ameliorating oxidative stress (Borrelli et al., 2018). It is certain that rMnSOD is full of potential in NASH treatment.

## 5 Treatment with microRNA

miRNAs are classes of endogenous non-coding RNAs consisting of about 22 nucleotides, activating or silencing target gene expression *via* regulating transcription and post-transcriptional processes, including the regulation of the non-coding RNA transcriptome, alternative mRNA splicing, translation, and mRNA degradation (Catalanotto et al., 2016). Thus, miRNAs are involved in multiple biological processes like cell proliferation, differentiation, apoptosis, tumorigenesis, and energy homeostasis (Jovanovic and Hengartner, 2006; Hwang and Mendell, 2007; Carrer et al., 2012).

The levels of hepatic miR-122 undergo a dynamic change in the downward trend during the progression of NAFLD to cirrhosis, and the ameliorative function of miR-122 on NASH is controversial (Hochreuter et al., 2022). Increased expression of PPARα and SIRT1 mediated by silencing miR-34a led to the improvement of hepatic steatosis and lipid metabolism (Ding et al., 2015). Lipophagy, mitochondrial biogenesis, and energy metabolism were inhibited following the activation of miR-34a and miR-let-7a mediated by LXRα (Kim et al., 2021b). There were no detectable NASH-related phenotypic alterations in normal/miR-21 knockout mice under normal dietary conditions, but miR-21 deficiency improved insulin resistance, steatosis, and lipid oxidation in mice with HFD, reducing inflammation and fibrosis in NASH (Calo et al., 2016; Loyer et al., 2016). Hepatocytes absorbed neutrophil-derived extracellular vesicles (EVs) filled with miR-223, mediated by a low-density lipoprotein receptor (LDLR) on hepatocytes and apolipoprotein E (APOE) on EVs, ameliorating hepatic inflammation and NASH in mice (He et al., 2021). The secretion of miR-223-enriched exosomes from macrophages facilitated by IL-6 altered gene expression in hepatocytes *via* an exosomal transfer (Hou et al., 2021). Kupffer cell-derived miR-690 also were shuttled to other cells like hepatocytes, recruited hepatic macrophages, and HSCs to improve insulin sensitivity, inflammation, and fibrosis in NASH mice through exosome-mediated transfer (Ying et al., 2021; Gao et al., 2022a). In addition to the aforementioned research, various miRNAs are involved in NASH progression.

Antagomirs are chemically engineered oligonucleotides silence-specific miRNAs *in vivo*, and they have been demonstrated as powerful tools to inhibit miRNA in diseases (Krützfeldt et al., 2005). The efficacy of miRNA-based therapy is primarily determined by effectively delivering miRNA or its inhibitor to targets. There are various oligonucleotide delivery strategies delivering drugs to specific cells and tissues, which comprise bioconjugation, lipid conjugates, liposomes, exosomes, and spherical nucleic acids (Roberts et al., 2020). Thus, miRNAs are highly promising in the treatment of NASH.

## 6 Therapeutic strategy using apoptosis inhibitors

Apoptosis is a strictly ordered programmed cell death characterized by programmed degradation of DNA, chromatin pyknosis, cell shrinkage, fragmentation, and apoptotic body formation (Kerr et al., 1972; Nagata, 2018), initiated by caspase (McIlwain et al., 2015). Apoptosis can be activated by a mitochondrial-dependent pathway and the extrinsic pathway mediated by TNF signaling pathway (Locksley et al., 2001; McIlwain et al., 2015). Apoptosis is closely related to the progression of NASH, and the role of inhibiting apoptosis in the treatment of NASH has also been confirmed (Kanda et al., 2018).

The TNF signaling pathway can cause apoptosis mainly depending on whether the anti-apoptotic signals such as NF-κB are inhibited (Tang et al., 2001). TNF-α induces the activation of apoptosis signal-regulating kinase 1 (ASK1), which activates p38/JUN N-terminal kinase (JNK) signaling, leading to apoptosis (De Smaele et al., 2001; Brenner et al., 2013). Cellular repressor of E1A-stimulated genes (CREG) and glutathione S-transferase Mu 2 (GSTM2) inhibits the phosphorylation of ASK1 to block its subsequent downstream signaling, thereby improving insulin resistance and hepatic steatosis (Zhang et al., 2017; Lan et al., 2022). The inhibition of ASK1-mediated activation of p38/JNK cascades delays the progression of NASH (Xiang et al., 2016). Some previous research studies have suggested that selonsertib (an inhibitor of ASK1) reduces fibrosis in NASH patients with fibrosis, whereas the improvement appears to be limited (Loomba et al., 2018; Harrison et al., 2020).

Caspase-8 plays a central role in apoptosis, which initiates apoptosis by activating caspase-3, caspase-6, and caspase-7 (Galluzzi et al., 2012; Yuan et al., 2016). A study showed that the ablation of caspase-8 protected against hepatic steatosis, accumulation of ROS, apoptosis, liver inflammation, and fibrosis in NASH mice (Hatting et al., 2013). In contrast, some studies have demonstrated that emricasan (a pan-caspase inhibitor) has no efficacy for NASH-related cirrhosis (Garcia-Tsao et al., 2020; Frenette et al., 2021). The possible reason for this result is that caspase-8 has a protective effect on preventing excess activation of necroptosis, and the key molecules of necroptosis may be the more potential targets for the treatment of NASH (Gautheron et al., 2014; Schwabe and Luedde, 2018). Consequently, the inhibition of RIPK1 which is the initiator of necroptosis has shown significantly therapeutic effects on NASH (Majdi et al., 2020).

## 7 Targeted therapeutic strategy of ferroptosis in NASH

Ferroptosis is an iron-dependent non-apoptotic form of cell death characterized by accumulation of reactive oxygen species (ROS) (Stockwell et al., 2017). Aberrant iron distribution leads to hepatic metabolic diseases. Hepatocytes have been confirmed to be iron deficient, and hepatic stellate cells have been demonstrated to be overloaded with iron in NAFLD (Gao et al., 2022b). Programmed cell death triggered by ROS accumulation in hepatocytes is a possible cause of liver injury and inflammation in NASH. Correspondingly, ferroptosis is the earliest form of cell death in NASH (Tsurusaki et al., 2019). Excess ferrous iron produces hydroxyl radicals through the Fenton reaction, thus leading to cytotoxicity including lipid peroxidation (Dixon et al., 2012; Jhelum et al., 2020). System x_c_^-^ (cystine/glutamate antiporter) promotes the uptake of cystine to increase the levels of glutathione (GSH), which is the main antioxidant *in vivo* (Mandal et al., 2010). The depletion of GSH indirectly leads to the inactivation of glutathione peroxidase 4 (GPX4), which causes disorders of lipid metabolism and ferroptosis (Yang et al., 2014). As discussed previously, iron disorders, lipid peroxidation, and disruption of the system x_c_^-^-GSH-GPX4 axis can trigger ferroptosis.

Serum ferritin (SF) levels are significantly increased in patients with NAFLD, which is considered to be an independent risk factor for the progression of NAFLD to NASH and fibrosis (Kowdley et al., 2012). Iron overload exacerbates insulin resistance associated with NASH (Fargion et al., 2005). In the methionine/choline-deficient diet (MCD) model, RSL-3 (a GPX4 inhibitor) aggravated liver steatosis, and inflammation in NASH, while deferoxamine mesylate nullified this effect through chelating iron. Treatment with GPX4 activator inhibited hepatic lipid peroxidation, while reducing the severity of NASH (Qi et al., 2020). Ferroptosis inhibition exhibits cytoprotection and alleviates inflammation in NASH (Tsurusaki et al., 2019). Iron accumulation in HSCs facilitates the progression of NASH to fibrosis *via* inducing fibrogenic activation, which is dependent on ROS (Gao et al., 2022b). As mentioned previously, targeting ferroptosis has significant potential for the prevention and treatment of NASH.

## 8 Stem cell-based therapy in NASH

**FIGURE 2:**
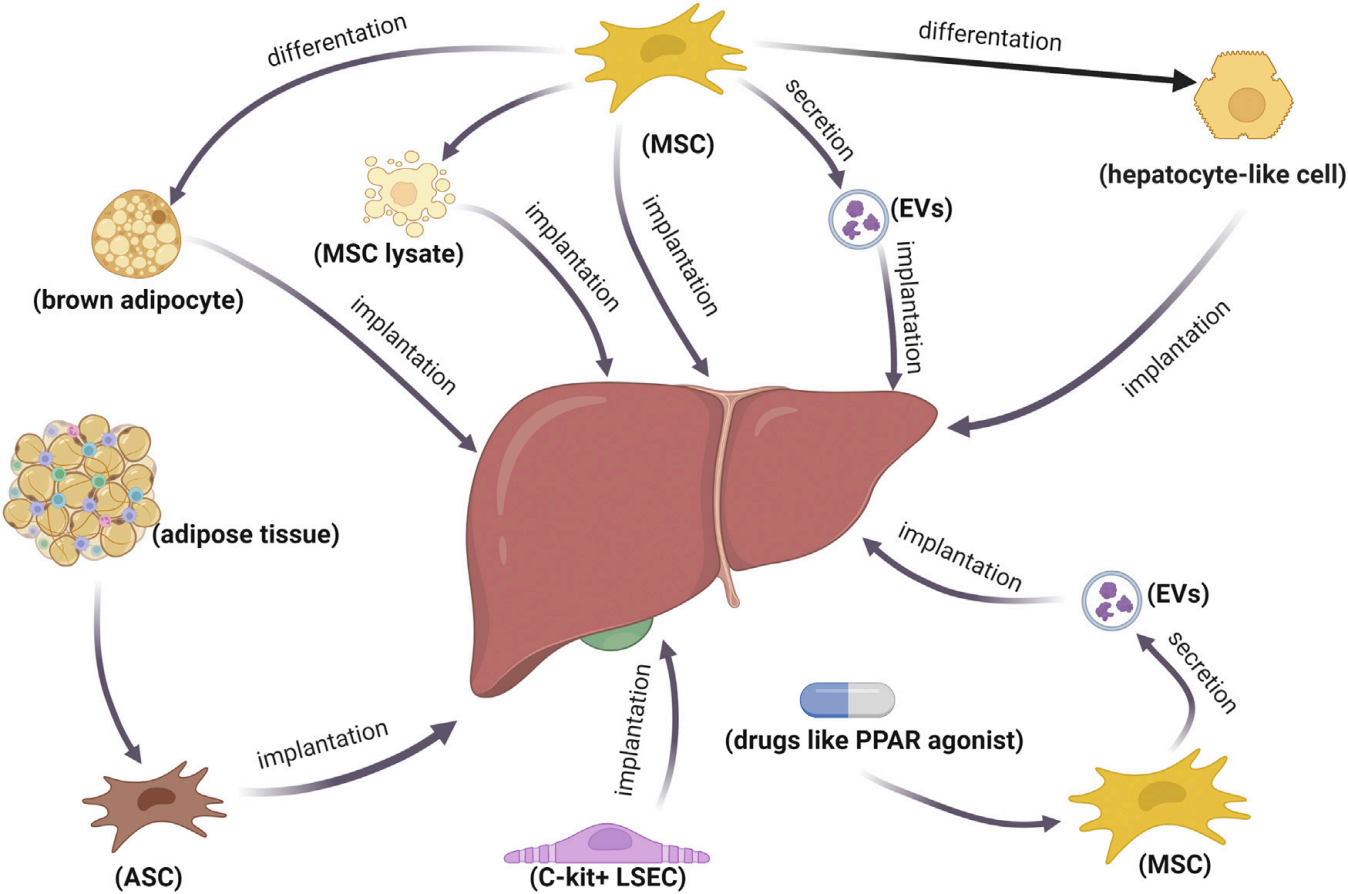
Therapeutic strategies of stem cells and their derivatives in NASH. Implantation of stem cells or stem-cell-differentiated cells is used to treat NASH. Additionally, stem cell derivatives are also promising for NASH treatment.

At present, cell transplantation for the treatment of metabolic diseases emerging from obesity has become a research hotspot. Mesenchymal stem cells (MSCs) are promising in the treatment of NASH, with extensive sources and rapid proliferation. The transplantation of MSC-derived human hepatocyte-like cells can restrain NASH progression by augmenting liver regeneration, inhibiting inflammation, and regulating lipid metabolism (Winkler et al., 2014). Intravenous infusion of adipose-derived mesenchymal stem cells (ASCs) improves insulin resistance through the hepatic AMPK signaling pathway and suppresses liver inflammation with decreasing TNFα (Cao et al., 2015; Xie et al., 2017). A recent study demonstrated that the infusion of c-kit-positive liver sinusoidal endothelial cells (LSECs) improves homeostasis in the pericentral liver endothelium and diet-induced NASH in mice by impacting LSEC–macrophage–neutrophil crosstalk mediated by repressing C-X-C motif chemokine receptor 4 (CXCR4) transcription by CCAAT enhancer-binding protein α (CEBPA) (Duan et al., 2022). In addition to cell implantation, EVs from pan-PPAR agonist-stimulated-induced mesenchymal stem cell (pan PPAR-iMSC-EVs) can facilitate NASH progression, contributing to ameliorating hepatic steatosis, ER stress, oxidative stress, and apoptosis (Kim et al., 2021a). MSC-derived apoptotic vesicles have significant potential in the treatment of NASH, considering macrophage regulatory effects and efferocytosis mediated by calreticulin (Zheng et al., 2021). Exosomes from human umbilical cord mesenchymal stem cells (hUC-MSCs) alleviated inflammation and reversed PPARα expression, thereby improving NASH in MCD-induced mice (Shi et al., 2022). MSC-based treatments regulate the expression of PPARα and PPARγ to reduce hepatic fat accumulation and mitigate NASH inflammation, among which MSC-derived brown adipocytes are more effective than MSCs and MSC lysate (Lee et al., 2017). Given that the safety, survival rate, and colonization rate of stem-cell transplantation are difficult to control, stem-cell derivatives may be more promising (Figure 2).

## 9 Targeted therapeutic agent delivery based on exosomes in NASH

Exosomes as a type of EVs are lipid-bilayer airtight vesicle with diameters ranging from 40 to 160 nm that are generated in a process that intraluminal vesicles (ILVs) are released to extracellular space upon fusion of intracellular multivesicular bodies (MVBs) containing ILVs and the plasma membrane (Gould and Raposo, 2013; Kalluri and LeBleu, 2020). Exosomes are naturally occurring targeted drug delivery tools *in vivo*, carrying proteins, lipids, nucleic acids, and synthetic drugs (Keerthikumar et al., 2016; Barile and Vassalli, 2017). Exosomes have exhibited multiple advantages in targeted drug delivery: 1) some exosomes can have a long retention time in circulation because CD47 protects exosomes from phagocytosis by macrophages and monocytes (Kamerkar et al., 2017); 2) there are abundant sources of exosomes; 3) exosomes are well-tolerated, less immunogenic, less antigenic, and less toxic (Kordelas et al., 2014; Zhu et al., 2017); 4) exosomes are able to migrate across biological barriers such as the blood–brain barrier with no modification (Yuan et al., 2017); 5) exosomes protect and maintain the biological activity of cargo, such as protecting exosomal RNA from degradation by ribonucleases present in blood (Cheng et al., 2014); and 6) surface modification of exosomes at the cellular level or after isolation can enhance targeting efficiency (Hood, 2016; Antimisiaris et al., 2018). In brief, exosome-mediated targeted therapy has fewer side effects, while regulating the targets more accurately to treat diseases.

The role of macrophages in NASH is so important that targeting macrophages is considered a promising therapeutic pathway (Huang et al., 2010; Kazankov et al., 2019). M2 macrophage polarization activated by a retinoic-acid-related orphan receptor α (RORα) contributes to the release of IL-10, which promotes M1 macrophage apoptosis, thus ameliorating NASH *via* inhibiting hepatic steatosis, inflammation, and apoptosis (Wan et al., 2014; Han et al., 2017). In the human body, exosomes from adipose-derived stem cells polarize M2 macrophages through the transactivation of arginase-1 by exosome-borne active STAT3 (Zhao et al., 2018). Further experimental evidence for exosome-mediated targeted drug delivery in NASH results from a study in which exosomes loaded with RBP-J decoy oligodeoxynucleotides block Notch signaling in macrophages and ameliorate liver inflammation (He et al., 2022). Exosomes are dominantly captured by macrophages *in vivo*, and exosomes of macrophages-targeted drug delivery do not appear to require a plethora of modifications (Imai et al., 2015).

As gatekeepers of liver homeostasis, LSECs make a significant contribution to the course of NAFLD/NASH (Hammoutene and Rautou, 2019). LSEC capillarization occurs at the early stage of NAFLD, defenestration, also called the loss of fenestrae aggravated in the cirrhotic phase (Miyao et al., 2015). In line with the aforementioned description, LSEC dysfunction occurs early in NAFLD, associated with augmented vascular resistance and steatosis (Pasarín et al., 2012; Nasiri-Ansari et al., 2022). LSECs without the physiological function promote pathologic angiogenesis, NASH progression, and fibrosis (Hammoutene and Rautou, 2019). Therapeutic approaches targeting LSECs are auspicious strategies for NASH treatment. The expression of Krüppel-like factor 2 (KLF2) activated by resveratrol or statins in LSECs repairs endothelial dysfunction while deactivating HSCs (Gracia-Sancho et al., 2010; Marrone et al., 2013). Furthermore, the blockade of endothelial Notch reverses LSEC dedifferentiation and improves LSEC angiocrine and NASH progression in an eNOS-dependent pathway (Duan et al., 2018; Fang et al., 2022). These are therapeutic targets for LSECs. How to treat NASH with targeting LSECs has been shown in a study that RUNX1 siRNA immunonano-lipocarrier tagged with VEGFR3 antibodies (specific marker of LSECs), specifically silenced the *RUNX1* gene in LSECs and improved NASH inflammation (Ding et al., 2010; Tripathi et al., 2021). Due to hyaluronic acid (HA) that is a naturally LSEC-targeted ligand, HA-coated nanocapsules efficiently deliver bioactive agents to LSECs (Fraser et al., 1985; Kren et al., 2009; Toriyabe et al., 2011). Moreover, the uptake efficiency of LSECs is affected by the density of ligands at the surface and the diameter of nanoparticles (Kamps et al., 1997; Akhter et al., 2014). Avoiding phagocytosis of macrophages is also the key to targeting LSEC by exosomes. CD47-enriched exosomes are possible to address this problem (Kamerkar et al., 2017; Belhadj et al., 2020). Otherwise, macrophage depletion by injection with clodronate-containing liposomes also delays the phagocytosis of exosomes by macrophages (Imai et al., 2015). As previously stated, exosomes of LSEC-targeted drug delivery are full of potential for NASH treatment.

**FIGURE 3:**
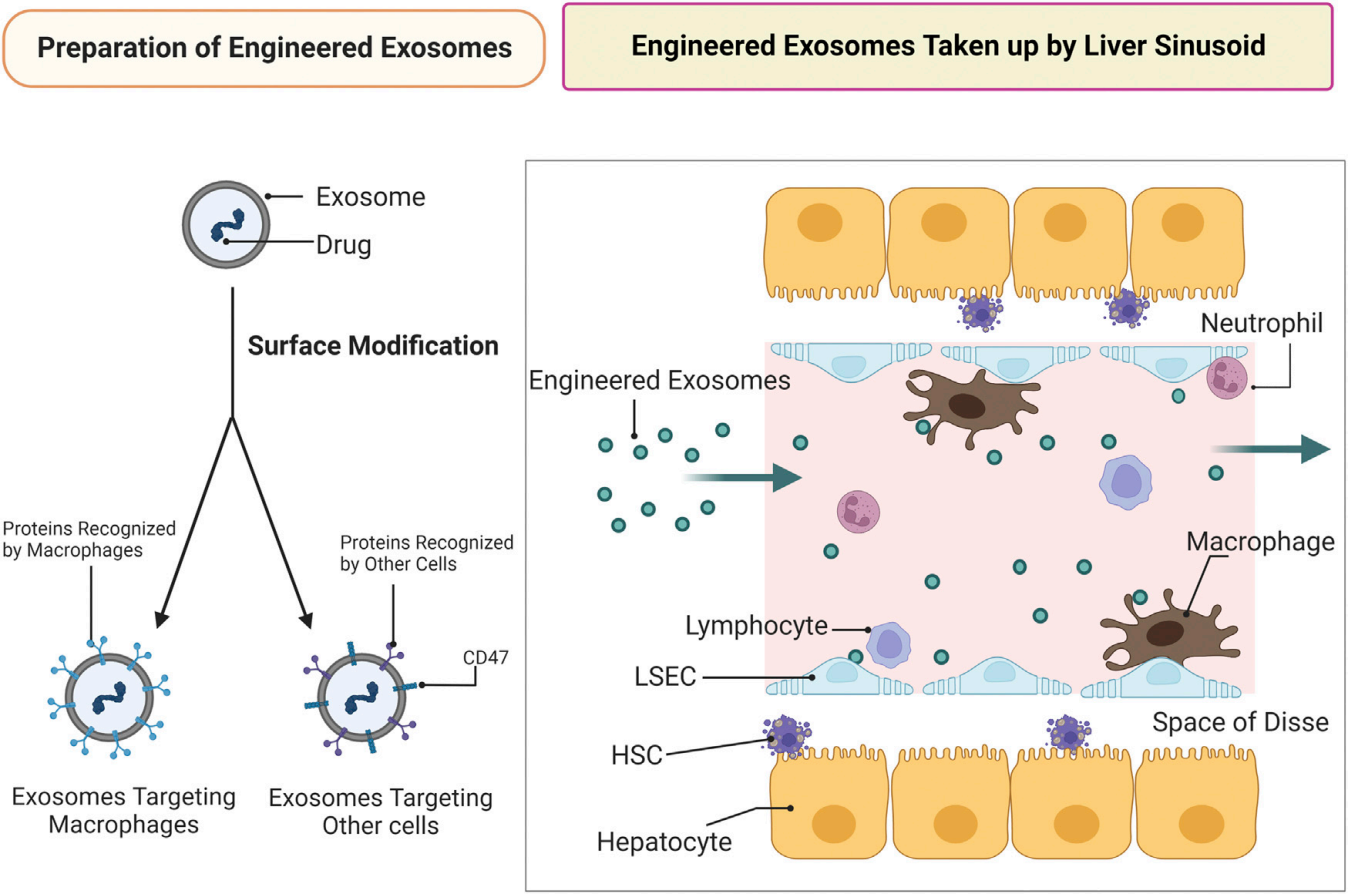
Engineered exosomes of targeted drug delivery against NASH. Preparation of engineered exosomes: drugs are to be loaded in the exosomes following exosomal surface modification. The targeting ability of engineered exosomes is enhanced by surface modification. Exosomes targeting cells other than macrophages require CD47 modification. Engineered exosomes taken up by liver sinusoid: infusion of engineered exosomes is used to treat NASH by delivering drugs to targets. Targeting cells other than LSECs and macrophages in therapeutic agents delivery is difficult due to hepatic histological characteristics and LSEC capillarization occurred at the early stage of NAFLD.

Apart from macrophages and LSECs, other cells in the liver may be therapeutic targets in the treatment of NASH. However, exosomes targeting other cells in therapeutic agent delivery are even more difficult based on hepatic histological characteristics (Figure 3).

## 10 Conclusion and perspective

Despite several drugs in late-stage clinical development, there is no existing therapy approved to treat NAFLD. Lifestyle intervention (diet, physical activity, and exercise) continues to be the mainstay of treatment (Romero-Gómez et al., 2017; Powell et al., 2021). NASH is a dynamic liver inflammatory response involving multiple metabolic disorders. In this review, the focus is placed on the summary of the role of metabolic regulation, the gut microbiome, antioxidant, miRNA, inhibition of apoptosis, ferroptosis resistance, and stem cell-based therapy in the management of NASH. As the therapeutic effect of single-targeted therapy was limited, combined therapy for distinct pathogenesis and targets and different periods exhibits better efficacy (Rajamoorthi et al., 2017; Puengel et al., 2022). This may increase the side effects of drugs. Precise targeted drug delivery such as engineered exosomes can use the aforementioned mechanisms and combat these problems. Exosome-mediated targeted drug delivery can be used to perform precise drug administration to selective targets with few adverse effects, according to the stage of development and pathological characteristics of NASH.

## References

[B1] Aithal G. P. Thomas J. A. Kaye P. V. Lawson A. Ryder S. D. Spendlove I. (2008). Randomized, placebo-controlled trial of pioglitazone in nondiabetic subjects with nonalcoholic steatohepatitis. Gastroenterology 135 (4), 1176–1184. 10.1053/j.gastro.2008.06.047 18718471

[B2] Akhter A. Hayashi Y. Sakurai Y. Ohga N. Hida K. Harashima H. (2014). Ligand density at the surface of a nanoparticle and different uptake mechanism: Two important factors for successful siRNA delivery to liver endothelial cells. Int. J. Pharm. 475 (1-2), 227–237. 10.1016/j.ijpharm.2014.08.048 25169077

[B3] Alonso C. Fernández-Ramos D. Varela-Rey M. Martínez-Arranz I. Navasa N. Van Liempd S. M. (2017). Metabolomic identification of subtypes of nonalcoholic steatohepatitis. Gastroenterology 152 (6), 1449–1461.e7. 10.1053/j.gastro.2017.01.015 28132890 PMC5406239

[B4] Antimisiaris S. G. Mourtas S. Marazioti A. (2018). Exosomes and exosome-inspired vesicles for targeted drug delivery. Pharmaceutics 10 (4), 218. 10.3390/pharmaceutics10040218 30404188 PMC6321407

[B5] Armstrong M. J. Gaunt P. Aithal G. P. Barton D. Hull D. Parker R. (2016). Liraglutide safety and efficacy in patients with non-alcoholic steatohepatitis (LEAN): A multicentre, double-blind, randomised, placebo-controlled phase 2 study. Lancet (London, Engl. 387 (10019), 679–690. 10.1016/S0140-6736(15)00803-X 26608256

[B6] Badman M. K. Pissios P. Kennedy A. R. Koukos G. Flier J. S. Maratos-Flier E. (2007). Hepatic fibroblast growth factor 21 is regulated by PPARalpha and is a key mediator of hepatic lipid metabolism in ketotic states. Cell Metab. 5 (6), 426–437. 10.1016/j.cmet.2007.05.002 17550778

[B7] Barile L. Vassalli G. (2017). Exosomes: Therapy delivery tools and biomarkers of diseases. Pharmacol. Ther. 174, 63–78. 10.1016/j.pharmthera.2017.02.020 28202367

[B8] Bates J. Vijayakumar A. Ghoshal S. Marchand B. Yi S. Kornyeyev D. (2020). Acetyl-CoA carboxylase inhibition disrupts metabolic reprogramming during hepatic stellate cell activation. J. Hepatology 73 (4), 896–905. 10.1016/j.jhep.2020.04.037 32376414

[B9] Beiroa D. Imbernon M. Gallego R. Senra A. Herranz D. Villarroya F. (2014). GLP-1 agonism stimulates Brown adipose tissue thermogenesis and browning through hypothalamic AMPK. Diabetes 63 (10), 3346–3358. 10.2337/db14-0302 24917578

[B10] Belfort R. Harrison S. A. Brown K. Darland C. Finch J. Hardies J. (2006). A placebo-controlled trial of pioglitazone in subjects with nonalcoholic steatohepatitis. N. Engl. J. Med. 355 (22), 2297–2307. 10.1056/NEJMoa060326 17135584

[B11] Belhadj Z. He B. Deng H. Song S. Zhang H. Wang X. (2020). A combined "eat me/don't eat me" strategy based on extracellular vesicles for anticancer nanomedicine. J. Extracell. Vesicles 9 (1), 1806444. 10.1080/20013078.2020.1806444 32944191 PMC7480498

[B12] Bohinc B. N. Michelotti G. Xie G. Pang H. Suzuki A. Guy C. D. (2014). Repair-related activation of hedgehog signaling in stromal cells promotes intrahepatic hypothyroidism. Endocrinology 155 (11), 4591–4601. 10.1210/en.2014-1302 25121996 PMC4256825

[B13] Borrelli A. Bonelli P. Tuccillo F. M. Goldfine I. D. Evans J. L. Buonaguro F. M. (2018). Role of gut microbiota and oxidative stress in the progression of non-alcoholic fatty liver disease to hepatocarcinoma: Current and innovative therapeutic approaches. Redox Biol. 15, 467–479. 10.1016/j.redox.2018.01.009 29413959 PMC5975181

[B14] Borrelli A. Schiattarella A. Mancini R. Pica A. Pollio M. L. Ruggiero M. G. (2016). A new hexapeptide from the leader peptide of rMnSOD enters cells through the oestrogen receptor to deliver therapeutic molecules. Sci. Rep. 6, 18691. 10.1038/srep18691 26725847 PMC4698655

[B15] Brenner C. Galluzzi L. Kepp O. Kroemer G. (2013). Decoding cell death signals in liver inflammation. J. Hepatology 59 (3), 583–594. 10.1016/j.jhep.2013.03.033 23567086

[B16] Brevini T. Maes M. Webb G. J. John B. V. Fuchs C. D. Buescher G. (2022). FXR inhibition may protect from SARS-CoV-2 infection by reducing ACE2. Nature 615, 134–142. 10.1038/s41586-022-05594-0 36470304 PMC9977684

[B17] Buzzetti E. Pinzani M. Tsochatzis E. A. (2016). The multiple-hit pathogenesis of non-alcoholic fatty liver disease (NAFLD). Metabolism Clin. Exp. 65 (8), 1038–1048. 10.1016/j.metabol.2015.12.012 26823198

[B18] Calle R. A. Amin N. B. Carvajal-Gonzalez S. Ross T. T. Bergman A. Aggarwal S. (2021). ACC inhibitor alone or co-administered with a DGAT2 inhibitor in patients with non-alcoholic fatty liver disease: Two parallel, placebo-controlled, randomized phase 2a trials. Nat. Med. 27 (10), 1836–1848. 10.1038/s41591-021-01489-1 34635855

[B19] Calo N. Ramadori P. Sobolewski C. Romero Y. Maeder C. Fournier M. (2016). Stress-activated *miR-21/miR-21** in hepatocytes promotes lipid and glucose metabolic disorders associated with high-fat diet consumption. Gut 65 (11), 1871–1881. 10.1136/gutjnl-2015-310822 27222533

[B20] Cao M. Pan Q. Dong H. Yuan X. Li Y. Sun Z. (2015). Adipose-derived mesenchymal stem cells improve glucose homeostasis in high-fat diet-induced obese mice. Stem Cell Res. Ther. 6, 208. 10.1186/s13287-015-0201-3 26519255 PMC4628312

[B21] Carpi R. Z. Barbalho S. M. Sloan K. P. Laurindo L. F. Gonzaga H. F. Grippa P. C. (2022). The effects of probiotics, Prebiotics and synbiotics in non-alcoholic fat liver disease (NAFLD) and non-alcoholic steatohepatitis (NASH): A systematic review. Int. J. Mol. Sci. 23 (15), 8805. 10.3390/ijms23158805 35955942 PMC9369010

[B22] Carrer M. Liu N. Grueter C. E. Williams A. H. Frisard M. I. Hulver M. W. (2012). Control of mitochondrial metabolism and systemic energy homeostasis by microRNAs 378 and 378*. Proc. Natl. Acad. Sci. U. S. A. 109 (38), 15330–15335. 10.1073/pnas.1207605109 22949648 PMC3458360

[B23] Catalanotto C. Cogoni C. Zardo G. (2016). MicroRNA in control of gene expression: An overview of nuclear functions. Int. J. Mol. Sci. 17 (10), 1712. 10.3390/ijms17101712 27754357 PMC5085744

[B24] Chau M. D. L. Gao J. Yang Q. Wu Z. Gromada J. (2010). Fibroblast growth factor 21 regulates energy metabolism by activating the AMPK-SIRT1-PGC-1alpha pathway. Proc. Natl. Acad. Sci. U. S. A. 107 (28), 12553–12558. 10.1073/pnas.1006962107 20616029 PMC2906565

[B25] Cheng L. Sharples R. A. Scicluna B. J. Hill A. F. (2014). Exosomes provide a protective and enriched source of miRNA for biomarker profiling compared to intracellular and cell-free blood. J. Extracell. Vesicles 3, 23743. 10.3402/jev.v3.23743 PMC3968297 24683445

[B26] Cobbold J. F. L. Atkinson S. Marchesi J. R. Smith A. Wai S. N. Stove J. (2018). Rifaximin in non-alcoholic steatohepatitis: An open-label pilot study. Hepatology Res. Official J. Jpn. Soc. Hepatology 48 (1), 69–77. 10.1111/hepr.12904 28425154

[B27] Coskun T. Sloop K. W. Loghin C. Alsina-Fernandez J. Urva S. Bokvist K. B. (2018). LY3298176, a novel dual GIP and GLP-1 receptor agonist for the treatment of type 2 diabetes mellitus: From discovery to clinical proof of concept. Mol. Metab. 18, 3–14. 10.1016/j.molmet.2018.09.009 30473097 PMC6308032

[B28] Craven L. Rahman A. Nair Parvathy S. Beaton M. Silverman J. Qumosani K. (2020). Allogenic fecal microbiota transplantation in patients with nonalcoholic fatty liver disease improves abnormal small intestinal permeability: A randomized control trial. Am. J. Gastroenterology 115 (7), 1055–1065. 10.14309/ajg.0000000000000661 32618656

[B29] Croasdell A. Duffney P. F. Kim N. Lacy S. H. Sime P. J. Phipps R. P. (2015). PPARγ and the innate immune system mediate the resolution of inflammation. PPAR Res. 2015, 549691. 10.1155/2015/549691 26713087 PMC4680113

[B30] Cunningham R. P. Moore M. P. Dashek R. J. Meers G. M. Takahashi T. Sheldon R. D. (2021). Critical role for hepatocyte-specific eNOS in NAFLD and NASH. Diabetes 70 (11), 2476–2491. 10.2337/db20-1228 34380696 PMC8564406

[B31] Das Pradhan A. Glynn R. J. Fruchart J.-C. MacFadyen J. G. Zaharris E. S. Everett B. M. (2022). Triglyceride lowering with pemafibrate to reduce cardiovascular risk. N. Engl. J. Med. 387 (21), 1923–1934. 10.1056/NEJMoa2210645 36342113

[B32] De Smaele E. Zazzeroni F. Papa S. Nguyen D. U. Jin R. Jones J. (2001). Induction of gadd45beta by NF-kappaB downregulates pro-apoptotic JNK signalling. Nature 414 (6861), 308–313. 10.1038/35104560 11713530

[B33] Ding B.-S. Nolan D. J. Butler J. M. James D. Babazadeh A. O. Rosenwaks Z. (2010). Inductive angiocrine signals from sinusoidal endothelium are required for liver regeneration. Nature 468 (7321), 310–315. 10.1038/nature09493 21068842 PMC3058628

[B34] Ding J. Li M. Wan X. Jin X. Chen S. Yu C. (2015). Effect of miR-34a in regulating steatosis by targeting PPARα expression in nonalcoholic fatty liver disease. Sci. Rep. 5, 13729. 10.1038/srep13729 26330104 PMC4557122

[B35] Dixon S. J. Lemberg K. M. Lamprecht M. R. Skouta R. Zaitsev E. M. Gleason C. E. (2012). Ferroptosis: An iron-dependent form of nonapoptotic cell death. Cell 149 (5), 1060–1072. 10.1016/j.cell.2012.03.042 22632970 PMC3367386

[B36] Dongiovanni P. Petta S. Mannisto V. Mancina R. M. Pipitone R. Karja V. (2015). Statin use and non-alcoholic steatohepatitis in at risk individuals. J. Hepatology 63 (3), 705–712. 10.1016/j.jhep.2015.05.006 25980762

[B37] Donkers J. M. Roscam Abbing R. L. P. van de Graaf S. F. J. (2019). Developments in bile salt based therapies: A critical overview. Biochem. Pharmacol. 161, 1–13. 10.1016/j.bcp.2018.12.018 30582898

[B38] Duan J.-L. Liu J.-J. Ruan B. Ding J. Fang Z.-Q. Xu H. (2022). Age-related liver endothelial zonation triggers steatohepatitis by inactivating pericentral endothelium-derived C-kit. Nat. Aging 3, 258–274. 10.1038/s43587-022-00348-z 37118422

[B39] Duan J.-L. Ruan B. Yan X.-C. Liang L. Song P. Yang Z.-Y. (2018). Endothelial Notch activation reshapes the angiocrine of sinusoidal endothelia to aggravate liver fibrosis and blunt regeneration in mice. Hepatology 68 (2), 677–690. 10.1002/hep.29834 29420858 PMC6099357

[B40] Erion M. D. Cable E. E. Ito B. R. Jiang H. Fujitaki J. M. Finn P. D. (2007). Targeting thyroid hormone receptor-beta agonists to the liver reduces cholesterol and triglycerides and improves the therapeutic index. Proc. Natl. Acad. Sci. U. S. A. 104 (39), 15490–15495. 10.1073/pnas.0702759104 17878314 PMC1978486

[B41] European Association for the Study of the Liver EASL. European Association for the Study of Diabetes EASD. European Association for the Study of Obesity EASO (2016). EASL-EASD-EASO Clinical Practice Guidelines for the management of non-alcoholic fatty liver disease. Diabetologia 59 (6), 1121–1140. 10.1007/s00125-016-3902-y 27053230

[B42] Fang Z.-Q. Ruan B. Liu J.-J. Duan J.-L. Yue Z.-S. Song P. (2022). Notch-triggered maladaptation of liver sinusoidal endothelium aggravates nonalcoholic steatohepatitis through endothelial nitric oxide synthase. Hepatology 76 (3), 742–758. 10.1002/hep.32332 35006626

[B43] Fargion S. Dongiovanni P. Guzzo A. Colombo S. Valenti L. Fracanzani A. L. (2005). Iron and insulin resistance. Alimentary Pharmacol. Ther. 22 (2), 61–63. 10.1111/j.1365-2036.2005.02599.x 16225476

[B44] Fiorucci S. Zampella A. Ricci P. Distrutti E. Biagioli M. (2022). Immunomodulatory functions of FXR. Mol. Cell. Endocrinol. 551, 111650. 10.1016/j.mce.2022.111650 35472625

[B45] Fisher F. M. Chui P. C. Nasser I. A. Popov Y. Cunniff J. C. Lundasen T. (2014). Fibroblast growth factor 21 limits lipotoxicity by promoting hepatic fatty acid activation in mice on methionine and choline-deficient diets. Gastroenterology 147 (5), 1073–1083.e6. 10.1053/j.gastro.2014.07.044 25083607 PMC4570569

[B46] Fougerat A. Montagner A. Loiseau N. Guillou H. Wahli W. (2020). Peroxisome proliferator-activated receptors and their novel ligands as candidates for the treatment of non-alcoholic fatty liver disease. Cells 9 (7), 1638. 10.3390/cells9071638 32650421 PMC7408116

[B47] Francque S. M. Bedossa P. Ratziu V. Anstee Q. M. Bugianesi E. Sanyal A. J. (2021). A randomized, controlled trial of the pan-PPAR agonist lanifibranor in NASH. N. Engl. J. Med. 385 (17), 1547–1558. 10.1056/NEJMoa2036205 34670042

[B48] Francque S. Verrijken A. Caron S. Prawitt J. Paumelle R. Derudas B. (2015). PPARα gene expression correlates with severity and histological treatment response in patients with non-alcoholic steatohepatitis. J. Hepatology 63 (1), 164–173. 10.1016/j.jhep.2015.02.019 25703085

[B49] Fraser J. R. Alcorn D. Laurent T. C. Robinson A. D. Ryan G. B. (1985). Uptake of circulating hyaluronic acid by the rat liver. Cellular localization *in situ* . Cell Tissue Res. 242 (3), 505–510. 10.1007/BF00225415 4075374

[B50] Frenette C. Kayali Z. Mena E. Mantry P. S. Lucas K. J. Neff G. (2021). Emricasan to prevent new decompensation in patients with NASH-related decompensated cirrhosis. J. Hepatology 74 (2), 274–282. 10.1016/j.jhep.2020.09.029 33038432

[B51] Galluzzi L. Vitale I. Abrams J. M. Alnemri E. S. Baehrecke E. H. Blagosklonny M. V. (2012). Molecular definitions of cell death subroutines: Recommendations of the nomenclature committee on cell death 2012. Cell Death Differ. 19 (1), 107–120. 10.1038/cdd.2011.96 21760595 PMC3252826

[B52] Gao H. Jin Z. Bandyopadhyay G. Cunha E Rocha K. Liu X. Zhao H. (2022a). MiR-690 treatment causes decreased fibrosis and steatosis and restores specific Kupffer cell functions in NASH. Cell Metab. 34 (7), 978–990.e4. 10.1016/j.cmet.2022.05.008 35700738 PMC9262870

[B53] Gao H. Jin Z. Bandyopadhyay G. Wang G. Zhang D. Rocha K. C. E. (2022b). Aberrant iron distribution via hepatocyte-stellate cell axis drives liver lipogenesis and fibrosis. Cell Metab. 34 (8), 1201–1213.e5. 10.1016/j.cmet.2022.07.006 35921818 PMC9365100

[B54] Garcia-Tsao G. Bosch J. Kayali Z. Harrison S. A. Abdelmalek M. F. Lawitz E. (2020). Randomized placebo-controlled trial of emricasan for non-alcoholic steatohepatitis-related cirrhosis with severe portal hypertension. J. Hepatology 72 (5), 885–895. 10.1016/j.jhep.2019.12.010 31870950

[B55] Gautheron J. Vucur M. Reisinger F. Cardenas D. V. Roderburg C. Koppe C. (2014). A positive feedback loop between RIP3 and JNK controls non-alcoholic steatohepatitis. EMBO Mol. Med. 6 (8), 1062–1074. 10.15252/emmm.201403856 24963148 PMC4154133

[B56] Gawrieh S. Noureddin M. Loo N. Mohseni R. Awasty V. Cusi K. (2021). Saroglitazar, a PPAR-α/γ agonist, for treatment of NAFLD: A randomized controlled double-blind phase 2 trial. Hepatology 74 (4), 1809–1824. 10.1002/hep.31843 33811367

[B57] Ginsberg H. N. Hounslow N. J. Senko Y. Suganami H. Bogdanski P. Ceska R. (2022). Efficacy and safety of K-877 (pemafibrate), a selective PPARα modulator, in European patients on statin therapy. Diabetes Care 45 (4), 898–908. 10.2337/dc21-1288 35238894

[B58] Gionfra F. De Vito P. Pallottini V. Lin H.-Y. Davis P. J. Pedersen J. Z. (2019). The role of thyroid hormones in hepatocyte proliferation and liver cancer. Front. Endocrinol. 10, 532. 10.3389/fendo.2019.00532 PMC6730500 31543862

[B59] Golabi P. Paik J. M. Eberly K. de Avila L. Alqahtani S. A. Younossi Z. M. (2022). Causes of death in patients with Non-alcoholic Fatty Liver Disease (NAFLD), alcoholic liver disease and chronic viral Hepatitis B and C. Ann. Hepatology 27 (1), 100556. 10.1016/j.aohep.2021.100556 34800721

[B60] Goldberg D. Ditah I. C. Saeian K. Lalehzari M. Aronsohn A. Gorospe E. C. (2017). Changes in the prevalence of hepatitis C virus infection, nonalcoholic steatohepatitis, and alcoholic liver disease among patients with cirrhosis or liver failure on the waitlist for liver transplantation. Gastroenterology 152 (5), 1090–1099.e1. 10.1053/j.gastro.2017.01.003 28088461 PMC5367965

[B61] Gould S. J. Raposo G. (2013). As we wait: Coping with an imperfect nomenclature for extracellular vesicles. J. Extracell. Vesicles 2, 20389. 10.3402/jev.v2i0.20389 PMC3760635 24009890

[B62] Gowda N. D Shiragannavar V. Lakshana D. P. Mathada R. Santhekadur P. (2022). Therapeutic role of saroglitazar in NAFLD and metabolic syndrome nov appro. Drug Des. Dev. 10.19080/NAPDD.2022.06.555689

[B63] Gracia-Sancho J. Villarreal G. Zhang Y. García-Cardeña G. (2010). Activation of SIRT1 by resveratrol induces KLF2 expression conferring an endothelial vasoprotective phenotype. Cardiovasc. Res. 85 (3), 514–519. 10.1093/cvr/cvp337 19815564 PMC2802207

[B64] Guillaume M. Rodriguez-Vilarrupla A. Gracia-Sancho J. Rosado E. Mancini A. Bosch J. (2013). Recombinant human manganese superoxide dismutase reduces liver fibrosis and portal pressure in CCl4-cirrhotic rats. J. Hepatology 58 (2), 240–246. 10.1016/j.jhep.2012.09.010 22989570

[B65] Hammoutene A. Rautou P.-E. (2019). Role of liver sinusoidal endothelial cells in non-alcoholic fatty liver disease. J. Hepatology 70 (6), 1278–1291. 10.1016/j.jhep.2019.02.012 30797053

[B66] Han Y.-H. Kim H.-J. Na H. Nam M.-W. Kim J.-Y. Kim J.-S. (2017). RORα induces KLF4-mediated M2 polarization in the liver macrophages that protect against nonalcoholic steatohepatitis. Cell Rep. 20 (1), 124–135. 10.1016/j.celrep.2017.06.017 28683306

[B67] Harriman G. Greenwood J. Bhat S. Huang X. Wang R. Paul D. (2016). Acetyl-CoA carboxylase inhibition by ND-630 reduces hepatic steatosis, improves insulin sensitivity, and modulates dyslipidemia in rats. Proc. Natl. Acad. Sci. U. S. A. 113 (13), E1796–E1805. 10.1073/pnas.1520686113 26976583 PMC4822632

[B68] Harrison S. A. Abdelmalek M. F. Neff G. Gunn N. Guy C. D. Alkhouri N. (2022). Aldafermin in patients with non-alcoholic steatohepatitis (ALPINE 2/3): A randomised, double-blind, placebo-controlled, phase 2b trial. Lancet. Gastroenterology Hepatology 7 (7), 603–616. 10.1016/S2468-1253(22)00017-6 35325622

[B69] Harrison S. A. Bashir M. R. Guy C. D. Zhou R. Moylan C. A. Frias J. P. (2019). Resmetirom (MGL-3196) for the treatment of non-alcoholic steatohepatitis: A multicentre, randomised, double-blind, placebo-controlled, phase 2 trial. Lancet (London, Engl. 394 (10213), 2012–2024. 10.1016/S0140-6736(19)32517-6 31727409

[B70] Harrison S. A. Neff G. Guy C. D. Bashir M. R. Paredes A. H. Frias J. P. (2021a). Efficacy and safety of aldafermin, an engineered FGF19 analog, in a randomized, double-blind, placebo-controlled trial of patients with nonalcoholic steatohepatitis. Gastroenterology 160 (1), 219–231.e1. 10.1053/j.gastro.2020.08.004 32781086

[B71] Harrison S. A. Ruane P. J. Freilich B. L. Neff G. Patil R. Behling C. A. (2021b). Efruxifermin in non-alcoholic steatohepatitis: A randomized, double-blind, placebo-controlled, phase 2a trial. Nat. Med. 27 (7), 1262–1271. 10.1038/s41591-021-01425-3 34239138

[B72] Harrison S. A. Wong V. W.-S. Okanoue T. Bzowej N. Vuppalanchi R. Younes Z. (2020). Selonsertib for patients with bridging fibrosis or compensated cirrhosis due to NASH: Results from randomized phase III STELLAR trials. J. Hepatology 73 (1), 26–39. 10.1016/j.jhep.2020.02.027 32147362

[B73] Hartman M. L. Sanyal A. J. Loomba R. Wilson J. M. Nikooienejad A. Bray R. (2020). Effects of novel dual GIP and GLP-1 receptor agonist tirzepatide on biomarkers of nonalcoholic steatohepatitis in patients with type 2 diabetes. Diabetes Care 43 (6), 1352–1355. 10.2337/dc19-1892 32291277 PMC7245348

[B74] Hatting M. Zhao G. Schumacher F. Sellge G. Al Masaoudi M. Gaβler N. (2013). Hepatocyte caspase-8 is an essential modulator of steatohepatitis in rodents. Hepatology 57 (6), 2189–2201. 10.1002/hep.26271 23339067

[B75] He F. Li W.-N. Li X.-X. Yue K.-Y. Duan J.-L. Ruan B. (2022). Exosome-mediated delivery of RBP-J decoy oligodeoxynucleotides ameliorates hepatic fibrosis in mice. Theranostics 12 (4), 1816–1828. 10.7150/thno.69885 35198075 PMC8825583

[B76] He Y. Rodrigues R. M. Wang X. Seo W. Ma J. Hwang S. (2021). Neutrophil-to-hepatocyte communication via LDLR-dependent miR-223-enriched extracellular vesicle transfer ameliorates nonalcoholic steatohepatitis. J. Clin. Investigation 131 (3), e141513. 10.1172/JCI141513 PMC7843220 33301423

[B77] Hill C. Guarner F. Reid G. Gibson G. R. Merenstein D. J. Pot B. (2014). Expert consensus document. The International Scientific Association for Probiotics and Prebiotics consensus statement on the scope and appropriate use of the term probiotic. Nat. Rev. Gastroenterology Hepatology 11 (8), 506–514. 10.1038/nrgastro.2014.66 24912386

[B78] Hochreuter M. Y. Dall M. Treebak J. T. Barrès R. (2022). MicroRNAs in non-alcoholic fatty liver disease: Progress and perspectives. Mol. Metab. 65, 101581. 10.1016/j.molmet.2022.101581 36028120 PMC9464960

[B79] Honda Y. Imajo K. Kato T. Kessoku T. Ogawa Y. Tomeno W. (2016). The selective SGLT2 inhibitor ipragliflozin has a therapeutic effect on nonalcoholic steatohepatitis in mice. PloS One 11 (1), e0146337. 10.1371/journal.pone.0146337 26731267 PMC4701474

[B80] Honda Y. Kessoku T. Ogawa Y. Tomeno W. Imajo K. Fujita K. (2017). Pemafibrate, a novel selective peroxisome proliferator-activated receptor alpha modulator, improves the pathogenesis in a rodent model of nonalcoholic steatohepatitis. Sci. Rep. 7, 42477. 10.1038/srep42477 28195199 PMC5307366

[B81] Hood J. L. (2016). Post isolation modification of exosomes for nanomedicine applications. Nanomedicine Lond. Engl. 11 (13), 1745–1756. 10.2217/nnm-2016-0102 PMC4941124 27348448

[B82] Hou X. Yin S. Ren R. Liu S. Yong L. Liu Y. (2021). Myeloid-cell-specific IL-6 signaling promotes MicroRNA-223-enriched exosome production to attenuate NAFLD-associated fibrosis. Hepatology 74 (1), 116–132. 10.1002/hep.31658 33236445 PMC8141545

[B83] Hu T. Chouinard M. Cox A. L. Sipes P. Marcelo M. Ficorilli J. (2006). Farnesoid X receptor agonist reduces serum asymmetric dimethylarginine levels through hepatic dimethylarginine dimethylaminohydrolase-1 gene regulation. J. Biol. Chem. 281 (52), 39831–39838. 10.1074/jbc.M606779200 17065154

[B84] Huang W. Metlakunta A. Dedousis N. Zhang P. Sipula I. Dube J. J. (2010). Depletion of liver Kupffer cells prevents the development of diet-induced hepatic steatosis and insulin resistance. Diabetes 59 (2), 347–357. 10.2337/db09-0016 19934001 PMC2809951

[B85] Hwang H. W. Mendell J. T. (2007). MicroRNAs in cell proliferation, cell death, and tumorigenesis. Br. J. Cancer 96, R40–R44. 10.1038/sj.bjc.6603023 17393584

[B86] Imai T. Takahashi Y. Nishikawa M. Kato K. Morishita M. Yamashita T. (2015). Macrophage-dependent clearance of systemically administered B16BL6-derived exosomes from the blood circulation in mice. J. Extracell. Vesicles 4, 26238. 10.3402/jev.v4.26238 25669322 PMC4323410

[B87] Iogna Prat L. Tsochatzis E. A. (2018). The effect of antidiabetic medications on non-alcoholic fatty liver disease (NAFLD). Horm. (Athens, Greece) 17 (2), 219–229. 10.1007/s42000-018-0021-9 29858843

[B88] Jain M. R. Giri S. R. Bhoi B. Trivedi C. Rath A. Rathod R. (2018). Dual PPARα/γ agonist saroglitazar improves liver histopathology and biochemistry in experimental NASH models. Liver Int. 38 (6), 1084–1094. 10.1111/liv.13634 29164820 PMC6001453

[B89] Jhelum P. Santos-Nogueira E. Teo W. Haumont A. Lenoël I. Stys P. K. (2020). Ferroptosis mediates cuprizone-induced loss of oligodendrocytes and demyelination. J. Neurosci. 40 (48), 9327–9341. 10.1523/JNEUROSCI.1749-20.2020 33106352 PMC7687057

[B90] Ji Y. Yin Y. Li Z. Zhang W. (2019). Gut microbiota-derived components and metabolites in the progression of non-alcoholic fatty liver disease (NAFLD). Nutrients 11 (8), 1712. 10.3390/nu11081712 31349604 PMC6724003

[B91] Jian J. Nie M.-T. Xiang B. Qian H. Yin C. Zhang X. (2022). Rifaximin ameliorates non-alcoholic steatohepatitis in mice through regulating gut microbiome-related bile acids. Front. Pharmacol. 13, 841132. 10.3389/fphar.2022.841132 35450049 PMC9017645

[B92] Jovanovic M. Hengartner M. O. (2006). miRNAs and apoptosis: RNAs to die for. Oncogene 25 (46), 6176–6187. 10.1038/sj.onc.1209912 17028597

[B93] Kabil S. L. Mahmoud N. M. (2018). Canagliflozin protects against non-alcoholic steatohepatitis in type-2 diabetic rats through zinc alpha-2 glycoprotein up-regulation. Eur. J. Pharmacol. 828, 135–145. 10.1016/j.ejphar.2018.03.043 29608898

[B94] Kaji K. Takaya H. Saikawa S. Furukawa M. Sato S. Kawaratani H. (2017). Rifaximin ameliorates hepatic encephalopathy and endotoxemia without affecting the gut microbiome diversity. World J. Gastroenterology 23 (47), 8355–8366. 10.3748/wjg.v23.i47.8355 PMC5743506 29307995

[B95] Kalluri R. LeBleu V. S. (2020). The biology function and biomedical applications of exosomes. Sci. (New York, N.Y.) 367 (6478), eaau6977. 10.1126/science.aau6977 PMC7717626 32029601

[B96] Kamerkar S. LeBleu V. S. Sugimoto H. Yang S. Ruivo C. F. Melo S. A. (2017). Exosomes facilitate therapeutic targeting of oncogenic KRAS in pancreatic cancer. Nature 546 (7659), 498–503. 10.1038/nature22341 28607485 PMC5538883

[B97] Kamps J. A. Morselt H. W. Swart P. J. Meijer D. K. Scherphof G. L. (1997). Massive targeting of liposomes, surface-modified with anionized albumins, to hepatic endothelial cells. Proc. Natl. Acad. Sci. U. S. A. 94 (21), 11681–11685. 10.1073/pnas.94.21.11681 9326670 PMC23586

[B98] Kanda T. Matsuoka S. Yamazaki M. Shibata T. Nirei K. Takahashi H. (2018). Apoptosis and non-alcoholic fatty liver diseases. World J. Gastroenterology 24 (25), 2661–2672. 10.3748/wjg.v24.i25.2661 PMC6034146 29991872

[B99] Kaneto H. Obata A. Shimoda M. Kimura T. Hirukawa H. Okauchi S. (2016). Promising diabetes therapy based on the molecular mechanism for glucose toxicity: Usefulness of SGLT2 inhibitors as well as incretin-related drugs. Curr. Med. Chem. 23 (27), 3044–3051. 10.2174/0929867323666160627102516 27356542

[B100] Kannt A. Wohlfart P. Madsen A. N. Veidal S. S. Feigh M. Schmoll D. (2021). Activation of thyroid hormone receptor-β improved disease activity and metabolism independent of body weight in a mouse model of non-alcoholic steatohepatitis and fibrosis. Br. J. Pharmacol. 178 (12), 2412–2423. 10.1111/bph.15427 33655500

[B101] Kazankov K. Jørgensen S. M. D. Thomsen K. L. Møller H. J. Vilstrup H. George J. (2019). The role of macrophages in nonalcoholic fatty liver disease and nonalcoholic steatohepatitis. Nat. Rev. Gastroenterology Hepatology 16 (3), 145–159. 10.1038/s41575-018-0082-x 30482910

[B102] Keerthikumar S. Chisanga D. Ariyaratne D. Al Saffar H. Anand S. Zhao K. (2016). ExoCarta: A web-based compendium of exosomal cargo. J. Mol. Biol. 428 (4), 688–692. 10.1016/j.jmb.2015.09.019 26434508 PMC4783248

[B103] Kerr J. F. Wyllie A. H. Currie A. R. (1972). Apoptosis: A basic biological phenomenon with wide-ranging implications in tissue kinetics. Br. J. Cancer 26 (4), 239–257. 10.1038/bjc.1972.33 4561027 PMC2008650

[B104] Khoruts A. (2018). Targeting the microbiome: From probiotics to fecal microbiota transplantation. Genome Med. 10 (1), 80. 10.1186/s13073-018-0592-8 30376869 PMC6208019

[B105] Kim C.-W. Addy C. Kusunoki J. Anderson N. N. Deja S. Fu X. (2017). Acetyl CoA carboxylase inhibition reduces hepatic steatosis but elevates plasma triglycerides in mice and humans: A bedside to bench investigation. Cell Metab. 26 (2), 394–406.e6. 10.1016/j.cmet.2017.07.009 28768177 PMC5603267

[B106] Kim J. Lee S. K. Jeong S.-Y. Cho H. J. Park J. Kim T. M. (2021a). Cargo proteins in extracellular vesicles: Potential for novel therapeutics in non-alcoholic steatohepatitis. J. Nanobiotechnology 19 (1), 372. 10.1186/s12951-021-01120-y 34789265 PMC8600817

[B107] Kim S. H. Kim K. H. Kim H.-K. Kim M.-J. Back S. H. Konishi M. (2015). Fibroblast growth factor 21 participates in adaptation to endoplasmic reticulum stress and attenuates obesity-induced hepatic metabolic stress. Diabetologia 58 (4), 809–818. 10.1007/s00125-014-3475-6 25537833

[B108] Kim Y. S. Nam H. J. Han C. Y. Joo M. S. Jang K. Jun D. W. (2021b). Liver X receptor alpha activation inhibits autophagy and Lipophagy in hepatocytes by dysregulating autophagy-related 4B cysteine peptidase and rab-8B, reducing mitochondrial fuel oxidation. Hepatology 73 (4), 1307–1326. 10.1002/hep.31423 32557804

[B109] Kir S. Beddow S. A. Samuel V. T. Miller P. Previs S. F. Suino-Powell K. (2011). FGF19 as a postprandial, insulin-independent activator of hepatic protein and glycogen synthesis. Sci. (New York, N.Y.) 331 (6024), 1621–1624. 10.1126/science.1198363 PMC3076083 21436455

[B110] Knudsen L. B. Lau J. (2019). The discovery and development of liraglutide and Semaglutide. Front. Endocrinol. 10, 155. 10.3389/fendo.2019.00155 PMC6474072 31031702

[B111] Kordelas L. Rebmann V. Ludwig A. K. Radtke S. Ruesing J. Doeppner T. R. (2014). MSC-Derived exosomes: A novel tool to treat therapy-refractory graft-versus-host disease. Leukemia 28 (4), 970–973. 10.1038/leu.2014.41 24445866

[B112] Kowdley K. V. Belt P. Wilson L. A. Yeh M. M. Neuschwander-Tetri B. A. Chalasani N. (2012). Serum ferritin is an independent predictor of histologic severity and advanced fibrosis in patients with nonalcoholic fatty liver disease. Hepatology 55 (1), 77–85. 10.1002/hep.24706 21953442 PMC3245347

[B113] Kren B. T. Unger G. M. Sjeklocha L. Trossen A. A. Korman V. Diethelm-Okita B. M. (2009). Nanocapsule-delivered Sleeping Beauty mediates therapeutic Factor VIII expression in liver sinusoidal endothelial cells of hemophilia A mice. J. Clin. Investigation 119 (7), 2086–2099. 10.1172/JCI34332 PMC2701853 19509468

[B114] Krützfeldt J. Rajewsky N. Braich R. Rajeev K. G. Tuschl T. Manoharan M. (2005). Silencing of microRNAs *in vivo* with 'antagomirs. Nature 438 (7068), 685–689. 10.1038/nature04303 16258535

[B115] Kuchay M. S. Krishan S. Mishra S. K. Farooqui K. J. Singh M. K. Wasir J. S. (2018). Effect of empagliflozin on liver fat in patients with type 2 diabetes and nonalcoholic fatty liver disease: A randomized controlled trial (E-LIFT trial). Diabetes Care 41 (8), 1801–1808. 10.2337/dc18-0165 29895557

[B116] Kumar D. P. Caffrey R. Marioneaux J. Santhekadur P. K. Bhat M. Alonso C. (2020). The PPAR α/γ agonist saroglitazar improves insulin resistance and steatohepatitis in a diet induced animal model of nonalcoholic fatty liver disease. Sci. Rep. 10 (1), 9330. 10.1038/s41598-020-66458-z 32518275 PMC7283326

[B117] Kurosu H. Choi M. Ogawa Y. Dickson A. S. Goetz R. Eliseenkova A. V. (2007). Tissue-specific expression of betaKlotho and fibroblast growth factor (FGF) receptor isoforms determines metabolic activity of FGF19 and FGF21. J. Biol. Chem. 282 (37), 26687–26695. 10.1074/jbc.M704165200 17623664 PMC2496965

[B118] Kwak M.-S. Kim D. Chung G. E. Kim W. Kim J. S. (2017). The preventive effect of sustained physical activity on incident nonalcoholic fatty liver disease. Liver Int. 37 (6), 919–926. 10.1111/liv.13332 27917585

[B119] Lalloyer F. Staels B. (2010). Fibrates, glitazones, and peroxisome proliferator-activated receptors. Arteriosclerosis, Thrombosis, Vasc. Biol. 30 (5), 894–899. 10.1161/ATVBAHA.108.179689 PMC2997800 20393155

[B120] Lan T. Hu Y. Hu F. Li H. Chen Y. Zhang J. (2022). Hepatocyte glutathione S-transferase mu 2 prevents non-alcoholic steatohepatitis by suppressing ASK1 signaling. J. Hepatology 76 (2), 407–419. 10.1016/j.jhep.2021.09.040 34656650

[B121] Lee C.-W. Hsiao W.-T. Lee O. K.-S. (2017). Mesenchymal stromal cell-based therapies reduce obesity and metabolic syndromes induced by a high-fat diet. Transl. Res. J. Laboratory Clin. Med. 182, 61–74.e8. 10.1016/j.trsl.2016.11.003 27908750

[B122] Lefere S. Puengel T. Hundertmark J. Penners C. Frank A. K. Guillot A. (2020). Differential effects of selective- and pan-PPAR agonists on experimental steatohepatitis and hepatic macrophages. J. Hepatology 73 (4), 757–770. 10.1016/j.jhep.2020.04.025 32360434

[B123] Li L. Zhang Q. Peng J. Jiang C. Zhang Y. Shen L. (2015). Activation of farnesoid X receptor downregulates monocyte chemoattractant protein-1 in murine macrophage. Biochem. Biophysical Res. Commun. 467 (4), 841–846. 10.1016/j.bbrc.2015.10.056 26474702

[B124] Lin B. C. Wang M. Blackmore C. Desnoyers L. R. (2007). Liver-specific activities of FGF19 require Klotho beta. J. Biol. Chem. 282 (37), 27277–27284. 10.1074/jbc.M704244200 17627937

[B125] Lin L. Zhang J. (2017). Role of intestinal microbiota and metabolites on gut homeostasis and human diseases. BMC Immunol. 18 (1), 2. 10.1186/s12865-016-0187-3 28061847 PMC5219689

[B126] Lin Z. Tian H. Lam K. S. L. Lin S. Hoo R. C. L. Konishi M. (2013). Adiponectin mediates the metabolic effects of FGF21 on glucose homeostasis and insulin sensitivity in mice. Cell Metab. 17 (5), 779–789. 10.1016/j.cmet.2013.04.005 23663741

[B127] Lippman S. M. Klein E. A. Goodman P. J. Lucia M. S. Thompson I. M. Ford L. G. (2009). Effect of selenium and vitamin E on risk of prostate cancer and other cancers: The selenium and vitamin E cancer prevention trial (SELECT). JAMA 301 (1), 39–51. 10.1001/jama.2008.864 19066370 PMC3682779

[B128] Liu J. Tian Y. Fu X. Mu C. Yao M. Ni Y. (2022). Estimating global prevalence, incidence, and outcomes of non-alcoholic fatty liver disease from 2000 to 2021: Systematic review and meta-analysis. Chin. Med. J. 135 (14), 1682–1691. 10.1097/CM9.0000000000002277 36070463 PMC9509027

[B129] Liu M. Zhang G. Wu S. Song M. Wang J. Cai W. (2020). Schaftoside alleviates HFD-induced hepatic lipid accumulation in mice via upregulating farnesoid X receptor. J. Ethnopharmacol. 255, 112776. 10.1016/j.jep.2020.112776 32205261

[B130] Locksley R. M. Killeen N. Lenardo M. J. (2001). The TNF and TNF receptor superfamilies: Integrating mammalian biology. Cell 104 (4), 487–501. 10.1016/s0092-8674(01)00237-9 11239407

[B131] Loomba R. Lawitz E. Mantry P. S. Jayakumar S. Caldwell S. H. Arnold H. (2018). The ASK1 inhibitor selonsertib in patients with nonalcoholic steatohepatitis: A randomized, phase 2 trial. Hepatology 67 (2), 549–559. 10.1002/hep.29514 28892558 PMC5814892

[B132] Loyer X. Paradis V. Hénique C. Vion A.-C. Colnot N. Guerin C. L. (2016). Liver microRNA-21 is overexpressed in non-alcoholic steatohepatitis and contributes to the disease in experimental models by inhibiting PPARα expression. Gut 65 (11), 1882–1894. 10.1136/gutjnl-2014-308883 26338827 PMC5099209

[B133] Lundåsen T. Gälman C. Angelin B. Rudling M. (2006). Circulating intestinal fibroblast growth factor 19 has a pronounced diurnal variation and modulates hepatic bile acid synthesis in man. J. Intern. Med. 260 (6), 530–536. 10.1111/j.1365-2796.2006.01731.x 17116003

[B134] Luo J. Sun P. Wang Y. Chen Y. Niu Y. Ding Y. (2021a). Dapagliflozin attenuates steatosis in livers of high-fat diet-induced mice and oleic acid-treated L02 cells via regulating AMPK/mTOR pathway. Eur. J. Pharmacol. 907, 174304. 10.1016/j.ejphar.2021.174304 34224699

[B135] Luo M. Yan J. Wu L. Wu J. Chen Z. Jiang J. (2021b). Probiotics alleviated nonalcoholic fatty liver disease in high-fat diet-fed rats via gut microbiota/FXR/FGF15 signaling pathway. J. Immunol. Res. 2021, 2264737. 10.1155/2021/2264737 34458376 PMC8387197

[B136] Ma J. Hennein R. Liu C. Long M. T. Hoffmann U. Jacques P. F. (2018). Improved diet quality associates with reduction in liver fat, particularly in individuals with high genetic risk scores for nonalcoholic fatty liver disease. Gastroenterology 155 (1), 107–117. 10.1053/j.gastro.2018.03.038 29604292 PMC6035111

[B137] Ma K. Saha P. K. Chan L. Moore D. D. (2006). Farnesoid X receptor is essential for normal glucose homeostasis. J. Clin. Investigation 116 (4), 1102–1109. 10.1172/JCI25604 PMC1409738 16557297

[B138] Majdi A. Aoudjehane L. Ratziu V. Islam T. Afonso M. B. Conti F. (2020). Inhibition of receptor-interacting protein kinase 1 improves experimental non-alcoholic fatty liver disease. J. Hepatology 72 (4), 627–635. 10.1016/j.jhep.2019.11.008 31760070

[B139] Mandal P. K. Seiler A. Perisic T. Kölle P. Banjac Canak A. Förster H. (2010). System x(c)- and thioredoxin reductase 1 cooperatively rescue glutathione deficiency. J. Biol. Chem. 285 (29), 22244–22253. 10.1074/jbc.M110.121327 20463017 PMC2903358

[B140] Mansouri R. M. Baugé E. Staels B. Gervois P. (2008). Systemic and distal repercussions of liver-specific peroxisome proliferator-activated receptor-alpha control of the acute-phase response. Endocrinology 149 (6), 3215–3223. 10.1210/en.2007-1339 18325987

[B141] Markova M. Pivovarova O. Hornemann S. Sucher S. Frahnow T. Wegner K. (2017). Isocaloric diets high in animal or plant protein reduce liver fat and inflammation in individuals with type 2 diabetes. Gastroenterology 152 (3), 571–585.e8. 10.1053/j.gastro.2016.10.007 27765690

[B142] Marrone G. Russo L. Rosado E. Hide D. García-Cardeña G. García-Pagán J. C. (2013). The transcription factor KLF2 mediates hepatic endothelial protection and paracrine endothelial-stellate cell deactivation induced by statins. J. Hepatology 58 (1), 98–103. 10.1016/j.jhep.2012.08.026 22989565

[B143] Masoodi M. Gastaldelli A. Hyötyläinen T. Arretxe E. Alonso C. Gaggini M. (2021). Metabolomics and lipidomics in NAFLD: Biomarkers and non-invasive diagnostic tests. Nat. Rev. Gastroenterology Hepatology 18 (12), 835–856. 10.1038/s41575-021-00502-9 34508238

[B144] McIlwain D. R. Berger T. Mak T. W. (2015). Caspase functions in cell death and disease. Cold Spring Harb. Perspect. Biol. 7 (4), a026716. 10.1101/cshperspect.a026716 25833847 PMC4382736

[B145] Miller E. R. Pastor-Barriuso R. Dalal D. Riemersma R. A. Appel L. J. Guallar E. (2005). Meta-analysis: high-dosage vitamin E supplementation may increase all-cause mortality. Ann. Intern. Med. 142 (1), 37–46. 10.7326/0003-4819-142-1-200501040-00110 15537682

[B146] Miyao M. Kotani H. Ishida T. Kawai C. Manabe S. Abiru H. (2015). Pivotal role of liver sinusoidal endothelial cells in NAFLD/NASH progression. Laboratory Investigation; a J. Tech. Methods Pathology 95 (10), 1130–1144. 10.1038/labinvest.2015.95 26214582

[B147] Mouries J. Brescia P. Silvestri A. Spadoni I. Sorribas M. Wiest R. (2019). Microbiota-driven gut vascular barrier disruption is a prerequisite for non-alcoholic steatohepatitis development. J. Hepatology 71 (6), 1216–1228. 10.1016/j.jhep.2019.08.005 PMC6880766 31419514

[B148] Mouzaki M. Comelli E. M. Arendt B. M. Bonengel J. Fung S. K. Fischer S. E. (2013). Intestinal microbiota in patients with nonalcoholic fatty liver disease. Hepatology 58 (1), 120–127. 10.1002/hep.26319 23401313

[B149] Mullard A. (2020). FDA rejects NASH drug. Nat. Rev. Drug Discov. 19 (8), 501. 10.1038/d41573-020-00126-9 32636504

[B150] Nagata S. (2018). Apoptosis and clearance of apoptotic cells. Annu. Rev. Immunol. 36, 489–517. 10.1146/annurev-immunol-042617-053010 29400998

[B151] Nakajima A. Eguchi Y. Yoneda M. Imajo K. Tamaki N. Suganami H. (2021). Randomised clinical trial: Pemafibrate, a novel selective peroxisome proliferator-activated receptor α modulator (SPPARMα), versus placebo in patients with non-alcoholic fatty liver disease. Alimentary Pharmacol. Ther. 54 (10), 1263–1277. 10.1111/apt.16596 PMC9292296 34528723

[B152] Nasiri-Ansari N. Androutsakos T. Flessa C.-M. Kyrou I. Siasos G. Randeva H. S. (2022). Endothelial cell dysfunction and nonalcoholic fatty liver disease (NAFLD): A concise review. Cells 11 (16), 2511. 10.3390/cells11162511 36010588 PMC9407007

[B153] Nasiri-Ansari N. Nikolopoulou C. Papoutsi K. Kyrou I. Mantzoros C. S. Kyriakopoulos G. (2021). Empagliflozin attenuates non-alcoholic fatty liver disease (NAFLD) in high fat diet fed ApoE mice by activating autophagy and reducing ER stress and apoptosis. Int. J. Mol. Sci. 22 (2), 818. 10.3390/ijms22020818 33467546 PMC7829901

[B154] Neuschwander-Tetri B. A. Caldwell S. H. (2003). Nonalcoholic steatohepatitis: Summary of an AASLD single topic conference. Hepatology 37 (5), 1202–1219. 10.1053/jhep.2003.50193 12717402

[B155] Neuschwander-Tetri B. A. Loomba R. Sanyal A. J. Lavine J. E. Van Natta M. L. Abdelmalek M. F. (2015). Farnesoid X nuclear receptor ligand obeticholic acid for non-cirrhotic, non-alcoholic steatohepatitis (FLINT): A multicentre, randomised, placebo-controlled trial. Lancet (London, Engl. 385 (9972), 956–965. 10.1016/S0140-6736(14)61933-4 PMC4447192 25468160

[B156] Newsome P. N. Buchholtz K. Cusi K. Linder M. Okanoue T. Ratziu V. (2021). A placebo-controlled trial of subcutaneous Semaglutide in nonalcoholic steatohepatitis. N. Engl. J. Med. 384 (12), 1113–1124. 10.1056/NEJMoa2028395 33185364

[B157] Ocker M. (2020). Fibroblast growth factor signaling in non-alcoholic fatty liver disease and non-alcoholic steatohepatitis: Paving the way to hepatocellular carcinoma. World J. Gastroenterology 26 (3), 279–290. 10.3748/wjg.v26.i3.279 PMC6969880 31988589

[B158] Okushin K. Tsutsumi T. Ikeuchi K. Kado A. Enooku K. Fujinaga H. (2020). Heterozygous knockout of Bile salt export pump ameliorates liver steatosis in mice fed a high-fat diet. PloS One 15 (8), e0234750. 10.1371/journal.pone.0234750 32785220 PMC7423142

[B159] Park E.-J. Lee Y.-S. Kim S. M. Park G.-S. Lee Y. H. Jeong D. Y. (2020). Beneficial effects of lactobacillus plantarum strains on non-alcoholic fatty liver disease in high fat/high fructose diet-fed rats. Nutrients 12 (2), 542. 10.3390/nu12020542 32093158 PMC7071439

[B160] Pasarín M. La Mura V. Gracia-Sancho J. García-Calderó H. Rodríguez-Vilarrupla A. García-Pagán J. C. (2012). Sinusoidal endothelial dysfunction precedes inflammation and fibrosis in a model of NAFLD. PloS One 7 (4), e32785. 10.1371/journal.pone.0032785 22509248 PMC3317918

[B161] Pellicciari R. Costantino G. Camaioni E. Sadeghpour B. M. Entrena A. Willson T. M. (2004). Bile acid derivatives as ligands of the farnesoid X receptor. Synthesis, evaluation, and structure-activity relationship of a series of body and side chain modified analogues of chenodeoxycholic acid. J. Med. Chem. 47 (18), 4559–4569. 10.1021/jm049904b 15317466

[B162] Piccinin E. Villani G. Moschetta A. (2019). Metabolic aspects in NAFLD, NASH and hepatocellular carcinoma: The role of PGC1 coactivators. Nat. Rev. Gastroenterology Hepatology 16 (3), 160–174. 10.1038/s41575-018-0089-3 30518830

[B163] Powell E. E. Wong V. W.-S. Rinella M. (2021). Non-alcoholic fatty liver disease. Lancet (London, Engl. 397 (10290), 2212–2224. 10.1016/S0140-6736(20)32511-3 33894145

[B164] Puengel T. Lefere S. Hundertmark J. Kohlhepp M. Penners C. Van de Velde F. (2022). Combined therapy with a CCR2/CCR5 antagonist and FGF21 analogue synergizes in ameliorating steatohepatitis and fibrosis. Int. J. Mol. Sci. 23 (12), 6696. 10.3390/ijms23126696 35743140 PMC9224277

[B165] Qi J. Kim J.-W. Zhou Z. Lim C.-W. Kim B. (2020). Ferroptosis affects the progression of nonalcoholic steatohepatitis via the modulation of lipid peroxidation-mediated cell death in mice. Am. J. Pathology 190 (1), 68–81. 10.1016/j.ajpath.2019.09.011 31610178

[B166] Qu S. Su D. Altomonte J. Kamagate A. He J. Perdomo G. (2007). PPAR{alpha} mediates the hypolipidemic action of fibrates by antagonizing FoxO1. Am. J. Physiology. Endocrinol. Metabolism 292 (2), E421–E434. 10.1152/ajpendo.00157.2006 PMC2665003 16985262

[B167] Rajamoorthi A. Arias N. Basta J. Lee R. G. Baldán Á. (2017). Amelioration of diet-induced steatohepatitis in mice following combined therapy with ASO-Fsp27 and fenofibrate. J. Lipid Res. 58 (11), 2127–2138. 10.1194/jlr.M077941 28874443 PMC5665668

[B168] Ratziu V. Charlotte F. Bernhardt C. Giral P. Halbron M. Lenaour G. (2010). Long-term efficacy of rosiglitazone in nonalcoholic steatohepatitis: Results of the fatty liver improvement by rosiglitazone therapy (FLIRT 2) extension trial. Hepatology 51 (2), 445–453. 10.1002/hep.23270 19877169

[B169] Ratziu V. Harrison S. A. Francque S. Bedossa P. Lehert P. Serfaty L. (2016). Elafibranor, an agonist of the peroxisome proliferator-activated receptor-α and -δ, induces resolution of nonalcoholic steatohepatitis without fibrosis worsening. Gastroenterology 150 (5), 1147–1159.e5. 10.1053/j.gastro.2016.01.038 26874076

[B170] Régnier M. Polizzi A. Smati S. Lukowicz C. Fougerat A. Lippi Y. (2020). Hepatocyte-specific deletion of Pparα promotes NAFLD in the context of obesity. Sci. Rep. 10 (1), 6489. 10.1038/s41598-020-63579-3 32300166 PMC7162950

[B171] Rieg T. Vallon V. (2018). Development of SGLT1 and SGLT2 inhibitors. Diabetologia 61 (10), 2079–2086. 10.1007/s00125-018-4654-7 30132033 PMC6124499

[B172] Roberts T. C. Langer R. Wood M. J. A. (2020). Advances in oligonucleotide drug delivery. Nat. Rev. Drug Discov. 19 (10), 673–694. 10.1038/s41573-020-0075-7 32782413 PMC7419031

[B173] Romero-Gómez M. Zelber-Sagi S. Trenell M. (2017). Treatment of NAFLD with diet, physical activity and exercise. J. Hepatology 67 (4), 829–846. 10.1016/j.jhep.2017.05.016 28545937

[B174] Ross T. T. Crowley C. Kelly K. L. Rinaldi A. Beebe D. A. Lech M. P. (2020). Acetyl-CoA carboxylase inhibition improves multiple dimensions of NASH pathogenesis in model systems. Cell. Mol. Gastroenterology Hepatology 10 (4), 829–851. 10.1016/j.jcmgh.2020.06.001 PMC7509217 32526482

[B175] Sanyal A. Charles E. D. Neuschwander-Tetri B. A. Loomba R. Harrison S. A. Abdelmalek M. F. (2019). Pegbelfermin (BMS-986036), a PEGylated fibroblast growth factor 21 analogue, in patients with non-alcoholic steatohepatitis: A randomised, double-blind, placebo-controlled, phase 2a trial. Lancet (London, Engl. 392 (10165), 2705–2717. 10.1016/S0140-6736(18)31785-9 30554783

[B176] Sanyal A. J. Chalasani N. Kowdley K. V. McCullough A. Diehl A. M. Bass N. M. (2010). Pioglitazone, vitamin E, or placebo for nonalcoholic steatohepatitis. N. Engl. J. Med. 362 (18), 1675–1685. 10.1056/NEJMoa0907929 20427778 PMC2928471

[B177] Sanyal A. J. (2019). Past, present and future perspectives in nonalcoholic fatty liver disease. Nat. Rev. Gastroenterology Hepatology 16 (6), 377–386. 10.1038/s41575-019-0144-8 31024089

[B178] Sasaki Y. Raza-Iqbal S. Tanaka T. Murakami K. Anai M. Osawa T. (2019). Gene expression profiles induced by a novel selective peroxisome proliferator-activated receptor α modulator (SPPARMα) pemafibrate. Int. J. Mol. Sci. 20 (22), 5682. 10.3390/ijms20225682 31766193 PMC6888257

[B179] Schmitt J. Kong B. Stieger B. Tschopp O. Schultze S. M. Rau M. (2015). Protective effects of farnesoid X receptor (FXR) on hepatic lipid accumulation are mediated by hepatic FXR and independent of intestinal FGF15 signal. Liver Int. 35 (4), 1133–1144. 10.1111/liv.12456 25156247 PMC4146754

[B180] Schürks M. Glynn R. J. Rist P. M. Tzourio C. Kurth T. (2010). Effects of vitamin E on stroke subtypes: meta-analysis of randomised controlled trials. BMJ Clin. Res. ed.) 341, c5702. 10.1136/bmj.c5702 PMC2974412 21051774

[B181] Schwabe R. F. Luedde T. (2018). Apoptosis and necroptosis in the liver: A matter of life and death. Nat. Rev. Gastroenterology Hepatology 15 (12), 738–752. 10.1038/s41575-018-0065-y 30250076 PMC6490680

[B182] Sekirov I. Russell S. L. Antunes L. C. M. Finlay B. B. (2010). Gut microbiota in health and disease. Physiol. Rev. 90 (3), 859–904. 10.1152/physrev.00045.2009 20664075

[B183] Setiawan V. W. Stram D. O. Porcel J. Lu S. C. Le Marchand L. Noureddin M. (2016). Prevalence of chronic liver disease and cirrhosis by underlying cause in understudied ethnic groups: The multiethnic cohort. Hepatology 64 (6), 1969–1977. 10.1002/hep.28677 27301913 PMC5115980

[B184] Shi Y. Yang X. Wang S. Wu Y. Zheng L. Tang Y. (2022). Human umbilical cord mesenchymal stromal cell-derived exosomes protect against MCD-induced NASH in a mouse model. Stem Cell Res. Ther. 13 (1), 517. 10.1186/s13287-022-03201-7 36371344 PMC9652856

[B185] Shiragannavar V. D. Sannappa Gowda N. G. Puttahanumantharayappa L. D. Karunakara S. H. Bhat S. S. Prasad S. (2023). The ameliorating effect of withaferin A on high-fat diet-induced non-alcoholic fatty liver disease by acting as an LXR/FXR dual receptor activator. Front. Pharmacol. 14, 1135952. 10.3389/fphar.2023.1135952 36909161 PMC9995434

[B186] Siddiqui M. S. Idowu M. O. Parmar D. Borg B. B. Denham D. Loo N. M. (2021). A phase 2 double blinded, randomized controlled trial of saroglitazar in patients with nonalcoholic steatohepatitis. Clin. Gastroenterology Hepatology 19 (12), 2670–2672. 10.1016/j.cgh.2020.10.051 33152542

[B187] Sinha R. A. Bruinstroop E. Singh B. K. Yen P. M. (2019). Nonalcoholic fatty liver disease and hypercholesterolemia: Roles of thyroid hormones, metabolites, and agonists. Thyroid 29 (9), 1173–1191. 10.1089/thy.2018.0664 31389309 PMC6850905

[B188] Sobhonslidsuk A. Chanprasertyothin S. Pongrujikorn T. Kaewduang P. Promson K. Petraksa S. (2018). The association of gut microbiota with nonalcoholic steatohepatitis in Thais. BioMed Res. Int. 2018, 9340316. 10.1155/2018/9340316 29682571 PMC5842744

[B189] Soden J. S. Devereaux M. W. Haas J. E. Gumpricht E. Dahl R. Gralla J. (2007). Subcutaneous vitamin E ameliorates liver injury in an *in vivo* model of steatocholestasis. Hepatology 46 (2), 485–495. 10.1002/hep.21690 17659596

[B190] Somm E. Jornayvaz F. R. (2018). Fibroblast growth factor 15/19: From basic functions to therapeutic perspectives. Endocr. Rev. 39 (6), 960–989. 10.1210/er.2018-00134 30124818

[B191] Song L. Wang L. Hou Y. Zhou J. Chen C. Ye X. (2022). FGF4 protects the liver from nonalcoholic fatty liver disease by activating the AMP-activated protein kinase-Caspase 6 signal axis. Hepatology 76 (4), 1105–1120. 10.1002/hep.32404 35152446

[B192] Stockwell B. R. Friedmann Angeli J. P. Bayir H. Bush A. I. Conrad M. Dixon S. J. (2017). Ferroptosis: A regulated cell death nexus linking metabolism, redox biology, and disease. Cell 171 (2), 273–285. 10.1016/j.cell.2017.09.021 28985560 PMC5685180

[B193] Sumida Y. Yoneda M. (2018). Current and future pharmacological therapies for NAFLD/NASH. J. Gastroenterology 53 (3), 362–376. 10.1007/s00535-017-1415-1 PMC5847174 29247356

[B194] Sun D. Zuo C. Huang W. Wang J. Zhang Z. (2022). Triclosan targeting of gut microbiome ameliorates hepatic steatosis in high fat diet-fed mice. J. Antibiotics 75 (6), 341–353. 10.1038/s41429-022-00522-w 35440769

[B195] Sung K.-C. Ryu S. Lee J.-Y. Kim J.-Y. Wild S. H. Byrne C. D. (2016). Effect of exercise on the development of new fatty liver and the resolution of existing fatty liver. J. Hepatology 65 (4), 791–797. 10.1016/j.jhep.2016.05.026 27255583

[B196] Tang G. Minemoto Y. Dibling B. Purcell N. H. Li Z. Karin M. (2001). Inhibition of JNK activation through NF-kappaB target genes. Nature 414 (6861), 313–317. 10.1038/35104568 11713531

[B197] Tang H. Shi W. Fu S. Wang T. Zhai S. Song Y. (2018). Pioglitazone and bladder cancer risk: A systematic review and meta-analysis. Cancer Med. 7 (4), 1070–1080. 10.1002/cam4.1354 29476615 PMC5911601

[B198] Teng Y. Zhao H. Gao L. Zhang W. Shull A. Y. Shay C. (2017). FGF19 protects hepatocellular carcinoma cells against endoplasmic reticulum stress via activation of FGFR4-gsk3β-nrf2 signaling. Cancer Res. 77 (22), 6215–6225. 10.1158/0008-5472.CAN-17-2039 28951455

[B199] Tilg H. Adolph T. E. Dudek M. Knolle P. (2021). Non-alcoholic fatty liver disease: The interplay between metabolism, microbes and immunity. Nat. Metab. 3 (12), 1596–1607. 10.1038/s42255-021-00501-9 34931080

[B200] Tomedi L. E. Roeber J. Landen M. (2018). Alcohol consumption and chronic liver disease mortality in new Mexico and the United States, 1999-2013. Public Health Rep. Wash. D.C. 1974) 133(3), 287–293. 10.1177/0033354918766890 PMC5958395 29664698

[B201] Toriyabe N. Hayashi Y. Hyodo M. Harashima H. (2011). Synthesis and evaluation of stearylated hyaluronic acid for the active delivery of liposomes to liver endothelial cells. Biol. Pharm. Bull. 34 (7), 1084–1089. 10.1248/bpb.34.1084 21720017

[B202] Tripathi D. M. Rohilla S. Kaur I. Siddiqui H. Rawal P. Juneja P. (2021). Immunonano-lipocarrier-mediated liver sinusoidal endothelial cell-specific RUNX1 inhibition impedes immune cell infiltration and hepatic inflammation in murine model of NASH. Int. J. Mol. Sci. 22 (16), 8489. 10.3390/ijms22168489 34445195 PMC8395158

[B203] Trovato F. M. Catalano D. Martines G. F. Pace P. Trovato G. M. (2015). Mediterranean diet and non-alcoholic fatty liver disease: The need of extended and comprehensive interventions. Clin. Nutr. Edinb. Scotl. 34 (1), 86–88. 10.1016/j.clnu.2014.01.018 24529325

[B204] Tsurusaki S. Tsuchiya Y. Koumura T. Nakasone M. Sakamoto T. Matsuoka M. (2019). Hepatic ferroptosis plays an important role as the trigger for initiating inflammation in nonalcoholic steatohepatitis. Cell Death Dis. 10 (6), 449. 10.1038/s41419-019-1678-y 31209199 PMC6579767

[B205] Tully D. C. Rucker P. V. Chianelli D. Williams J. Vidal A. Alper P. B. (2017). Discovery of tropifexor (LJN452), a highly potent non-bile acid FXR agonist for the treatment of cholestatic liver diseases and nonalcoholic steatohepatitis (NASH). J. Med. Chem. 60 (24), 9960–9973. 10.1021/acs.jmedchem.7b00907 29148806

[B206] Tziomalos K. Athyros V. G. Paschos P. Karagiannis A. (2015). Nonalcoholic fatty liver disease and statins. Metabolism Clin. Exp. 64 (10), 1215–1223. 10.1016/j.metabol.2015.07.003 26234727

[B207] Velkov T. (2013). Interactions between human liver fatty acid binding protein and peroxisome proliferator activated receptor selective drugs. PPAR Res. 2013, 938401. 10.1155/2013/938401 23476633 PMC3588188

[B208] Vilar-Gomez E. Martinez-Perez Y. Calzadilla-Bertot L. Torres-Gonzalez A. Gra-Oramas B. Gonzalez-Fabian L. (2015). Weight loss through Lifestyle modification significantly reduces features of nonalcoholic steatohepatitis. Gastroenterology 149 (2), 367–378.e5. 10.1053/j.gastro.2015.04.005 25865049

[B209] Wan J. Benkdane M. Teixeira-Clerc F. Bonnafous S. Louvet A. Lafdil F. (2014). M2 kupffer cells promote M1 kupffer cell apoptosis: A protective mechanism against alcoholic and nonalcoholic fatty liver disease. Hepatology 59 (1), 130–142. 10.1002/hep.26607 23832548

[B210] Wang Y.-D. Chen W.-D. Wang M. Yu D. Forman B. M. Huang W. (2008). Farnesoid X receptor antagonizes nuclear factor kappaB in hepatic inflammatory response. Hepatology 48 (5), 1632–1643. 10.1002/hep.22519 18972444 PMC3056574

[B211] Winkler S. Borkham-Kamphorst E. Stock P. Brückner S. Dollinger M. Weiskirchen R. (2014). Human mesenchymal stem cells towards non-alcoholic steatohepatitis in an immunodeficient mouse model. Exp. Cell Res. 326 (2), 230–239. 10.1016/j.yexcr.2014.04.017 24786317

[B212] Wong V. W.-S. Tse C.-H. Lam T. T.-Y. Wong G. L.-H. Chim A. M.-L. Chu W. C.-W. (2013). Molecular characterization of the fecal microbiota in patients with nonalcoholic steatohepatitis-a longitudinal study. PloS One 8 (4), e62885. 10.1371/journal.pone.0062885 23638162 PMC3636208

[B213] Xiang M. Wang P.-X. Wang A.-B. Zhang X.-J. Zhang Y. Zhang P. (2016). Targeting hepatic TRAF1-ASK1 signaling to improve inflammation, insulin resistance, and hepatic steatosis. J. Hepatology 64 (6), 1365–1377. 10.1016/j.jhep.2016.02.002 26860405

[B214] Xie M. Hao H. J. Cheng Y. Xie Z. Y. Yin Y. Q. Zhang Q. (2017). Adipose-derived mesenchymal stem cells ameliorate hyperglycemia through regulating hepatic glucose metabolism in type 2 diabetic rats. Biochem. Biophysical Res. Commun. 483 (1), 435–441. 10.1016/j.bbrc.2016.12.125 28013047

[B215] Yang W. S. SriRamaratnam R. Welsch M. E. Shimada K. Skouta R. Viswanathan V. S. (2014). Regulation of ferroptotic cancer cell death by GPX4. Cell 156 (1-2), 317–331. 10.1016/j.cell.2013.12.010 24439385 PMC4076414

[B216] Ying W. Gao H. Dos Reis F. C. G. Bandyopadhyay G. Ofrecio J. M. Luo Z. (2021). MiR-690, an exosomal-derived miRNA from M2-polarized macrophages, improves insulin sensitivity in obese mice. Cell Metab. 33 (4), 781–790.e5. 10.1016/j.cmet.2020.12.019 33450179 PMC8035248

[B217] Yki-Järvinen H. (2014). Non-alcoholic fatty liver disease as a cause and a consequence of metabolic syndrome. Lancet. Diabetes and Endocrinol. 2 (11), 901–910. 10.1016/S2213-8587(14)70032-4 24731669

[B218] Younossi Z. Anstee Q. M. Marietti M. Hardy T. Henry L. Eslam M. (2018). Global burden of NAFLD and NASH: Trends, predictions, risk factors and prevention. Nat. Rev. Gastroenterology Hepatology 15 (1), 11–20. 10.1038/nrgastro.2017.109 28930295

[B219] Younossi Z. M. Koenig A. B. Abdelatif D. Fazel Y. Henry L. Wymer M. (2016). Global epidemiology of nonalcoholic fatty liver disease-Meta-analytic assessment of prevalence, incidence, and outcomes. Hepatology 64 (1), 73–84. 10.1002/hep.28431 26707365

[B220] Younossi Z. M. Ratziu V. Loomba R. Rinella M. Anstee Q. M. Goodman Z. (2019). Obeticholic acid for the treatment of non-alcoholic steatohepatitis: Interim analysis from a multicentre, randomised, placebo-controlled phase 3 trial. Lancet (London, Engl. 394 (10215), 2184–2196. 10.1016/S0140-6736(19)33041-7 31813633

[B221] Yuan D. Zhao Y. Banks W. A. Bullock K. M. Haney M. Batrakova E. (2017). Macrophage exosomes as natural nanocarriers for protein delivery to inflamed brain. Biomaterials 142, 1–12. 10.1016/j.biomaterials.2017.07.011 28715655 PMC5603188

[B222] Yuan J. Najafov A. Py B. F. (2016). Roles of caspases in necrotic cell death. Cell 167 (7), 1693–1704. 10.1016/j.cell.2016.11.047 27984721 PMC5381727

[B223] Zhang Q.-Y. Zhao L.-P. Tian X.-X. Yan C.-H. Li Y. Liu Y.-X. (2017). The novel intracellular protein CREG inhibits hepatic steatosis, obesity, and insulin resistance. Hepatology 66 (3), 834–854. 10.1002/hep.29257 28508477

[B224] Zhang X.-J. Ji Y.-X. Cheng X. Cheng Y. Yang H. Wang J. (2021a). A small molecule targeting ALOX12-ACC1 ameliorates nonalcoholic steatohepatitis in mice and macaques. Sci. Transl. Med. 13 (624), eabg8116. 10.1126/scitranslmed.abg8116 34910548

[B225] Zhang X.-J. She Z.-G. Wang J. Sun D. Shen L.-J. Xiang H. (2021b). Multiple omics study identifies an interspecies conserved driver for nonalcoholic steatohepatitis. Sci. Transl. Med. 13 (624), eabg8117. 10.1126/scitranslmed.abg8117 34910546

[B226] Zhao H. Shang Q. Pan Z. Bai Y. Li Z. Zhang H. (2018). Exosomes from adipose-derived stem cells attenuate adipose inflammation and obesity through polarizing M2 macrophages and beiging in white adipose tissue. Diabetes 67 (2), 235–247. 10.2337/db17-0356 29133512

[B227] Zheng C. Sui B. Zhang X. Hu J. Chen J. Liu J. (2021). Apoptotic vesicles restore liver macrophage homeostasis to counteract type 2 diabetes. J. Extracell. Vesicles 10 (7), e12109. 10.1002/jev2.12109 34084287 PMC8144839

[B228] Zhou D. Pan Q. Shen F. Cao H.-X. Ding W.-J. Chen Y.-W. (2017). Total fecal microbiota transplantation alleviates high-fat diet-induced steatohepatitis in mice via beneficial regulation of gut microbiota. Sci. Rep. 7 (1), 1529. 10.1038/s41598-017-01751-y 28484247 PMC5431549

[B229] Zhou M. Learned R. M. Rossi S. J. DePaoli A. M. Tian H. Ling L. (2016). Engineered fibroblast growth factor 19 reduces liver injury and resolves sclerosing cholangitis in Mdr2-deficient mice. Hepatology 63 (3), 914–929. 10.1002/hep.28257 26418580 PMC5063176

[B230] Zhu X. Badawi M. Pomeroy S. Sutaria D. S. Xie Z. Baek A. (2017). Comprehensive toxicity and immunogenicity studies reveal minimal effects in mice following sustained dosing of extracellular vesicles derived from HEK293T cells. J. Extracell. Vesicles 6 (1), 1324730. 10.1080/20013078.2017.1324730 28717420 PMC5505007

[B231] Zhuang L.-n. Hu W.-x. Xin S.-m. Zhao J. Pei G. (2011). Beta-arrestin-1 protein represses adipogenesis and inflammatory responses through its interaction with peroxisome proliferator-activated receptor-gamma (PPARgamma). J. Biol. Chem. 286 (32), 28403–28413. 10.1074/jbc.M111.256099 21700709 PMC3151083

